# Chemokine (C-C Motif) Receptor 2 Mediates Dendritic Cell Recruitment to the Human Colon but Is Not Responsible for Differences Observed in Dendritic Cell Subsets, Phenotype, and Function Between the Proximal and Distal Colon

**DOI:** 10.1016/j.jcmgh.2015.08.006

**Published:** 2015-09-03

**Authors:** David Bernardo, Lydia Durant, Elizabeth R. Mann, Elizabeth Bassity, Enrique Montalvillo, Ripple Man, Rakesh Vora, Durga Reddi, Fahri Bayiroglu, Luis Fernández-Salazar, Nick R. English, Simon T.C. Peake, Jon Landy, Gui H. Lee, George Malietzis, Yi Harn Siaw, Aravinth U. Murugananthan, Phil Hendy, Eva Sánchez-Recio, Robin K.S. Phillips, Jose A. Garrote, Paul Scott, Julian Parkhill, Malte Paulsen, Ailsa L. Hart, Hafid O. Al-Hassi, Eduardo Arranz, Alan W. Walker, Simon R. Carding, Stella C. Knight

**Affiliations:** 1 Antigen Presentation Research Group, Imperial College London, Harrow, United Kingdom; 2 Centre for Immunobiology, Institute of Infection, Immunity and Inflammation, University of Glasgow, Glasgow, United Kingdom; 3 Gut Health and Food Safety Programme, Institute of Food Research, Norwich, United Kingdom; 4 Mucosal Immunology Group, Instituto de Biología y Genética Molecular (IBGM), Universidad de Valladolid–CSIC, Valladolid, Spain; 5 St. Mark’s Hospital, North West London Hospitals NHS Trust, Harrow, United Kingdom; 6 Department of Physiology, Faculty of Medicine, Yildirim Beyazit University, Ankara, Turkey; 7 Faculty of Farmacy, Agri İbrahim Cecen University, Agri, Turkey; 8 Gastroenterology Service, Hospital Clínico Universitario de Valladolid, Valladolid, Spain; 9 Genetics and Molecular Biology Department, Clinical Laboratory Service, Hospital Universitario Rio Hortega, Valladolid, Spain; 10 Pathogen Genomics Group, Wellcome Trust Sanger Institute, Wellcome Trust Genome Campus, Hinxton, Cambridgeshire, United Kingdom; 11 National Heart and Lung Institute, Imperial College London, London; 12 Microbiology Group, Rowett Institute of Nutrition and Health, University of Aberdeen, Aberdeen, United Kingdom; 13 Norwich Medical School, University of East Anglia, Norwich, United Kingdom

**Keywords:** CCR2, Dendritic Cells, Distal Colon, Human Gastrointestinal Tract, Proximal Colon, Microbiota, AMOVA, analysis of molecular variance, CCL, chemokine (C-C motif) ligand, CCR, chemokine (C-C motif) receptor, CFSE, 5-carboxy fluorescein diacetate succinimidyl ester, DC, dendritic cells, DL, detection limit, FACS, fluorescence-activated cell sorting, FITC, fluorescein isothiocyanate, GI, gastrointestinal, IL, interleukin, ILT3, Ig-like transcript 3, LPMC, lamina propria mononuclear cells, Mφ, macrophages, mDC, myeloid dendritic cell, PBMC, peripheral blood mononuclear cells, PCR, polymerase chain reaction, pDC, plasmacytoid dendritic cell, RALDH2, retinaldehyde dehydrogenase type 2, SIRPα, signal regulatory protein α, SPB, sodium phosphate buffer, Treg, regulatory T-cells

## Abstract

**Background & Aims:**

Most knowledge about gastrointestinal (GI)-tract dendritic cells (DC) relies on murine studies where CD103^+^ DC specialize in generating immune tolerance with the functionality of CD11b^+/−^ subsets being unclear. Information about human GI-DC is scarce, especially regarding regional specifications. Here, we characterized human DC properties throughout the human colon.

**Methods:**

Paired proximal (right/ascending) and distal (left/descending) human colonic biopsies from 95 healthy subjects were taken; DC were assessed by flow cytometry and microbiota composition assessed by 16S rRNA gene sequencing.

**Results:**

Colonic DC identified were myeloid (mDC, CD11c^+^CD123^−^) and further divided based on CD103 and SIRPα (human analog of murine CD11b) expression. CD103^-^SIRPα^+^ DC were the major population and with CD103^+^SIRPα^+^ DC were CD1c^+^ILT3^+^CCR2^+^ (although CCR2 was not expressed on all CD103^+^SIRPα^+^ DC). CD103^+^SIRPα^-^ DC constituted a minor subset that were CD141^+^ILT3^−^CCR2^−^. Proximal colon samples had higher total DC counts and fewer CD103^+^SIRPα^+^ cells. Proximal colon DC were more mature than distal DC with higher stimulatory capacity for CD4^+^CD45RA^+^ T-cells. However, DC and DC-invoked T-cell expression of mucosal homing markers (β7, CCR9) was lower for proximal DC. CCR2 was expressed on circulating CD1c^+^, but not CD141^+^ mDC, and mediated DC recruitment by colonic culture supernatants in transwell assays. Proximal colon DC produced higher levels of cytokines. Mucosal microbiota profiling showed a lower microbiota load in the proximal colon, but with no differences in microbiota composition between compartments.

**Conclusions:**

Proximal colonic DC subsets differ from those in distal colon and are more mature. Targeted immunotherapy using DC in T-cell mediated GI tract inflammation may therefore need to reflect this immune compartmentalization.

**Summary**

Intestinal dendritic cells (DC) maintain a balance between tolerance of nutrients/commensals and immunity against pathogens. Here, we report lower numbers of CD103^+^SIRPα^+^ DC, with a more mature phenotype and higher immune activity, in the proximal than in the distal healthy human colon.

Dendritic cells (DC), unique in their ability to generate primary T-cell-mediated immune responses and potent promoters of secondary activity, dictate whether immune responses are immunogenic or tolerogenic and determine their tissue specificity.^1^ In the gastrointestinal (GI) tract, DC maintain a balance between tolerance to nutrients and/or commensal microorganisms (the microbiota) and immunity against pathogens.^2^, ^3^, ^4^

Human myeloid DC (mDC; CD11c^+^) can be divided into CD1c^+^ (analogous to mouse CD11b^+^ specialized for classic presentation) and CD141^+^ (analogous to mouse CD8α^+^ specialized for cross presentation).^5^ Most current knowledge about GI tract DC comes from mice, and typically from the small intestine, where CD103^+^ DC specialize in generating regulatory T-cell (Treg) responses critical for prevention of spontaneous inflammation. Some of these properties appear to be maintained in the human GI tract.^6^, ^7^, ^8^ Further characterization of murine CD103^+^ DC revealed that the subset coexpressing CD11b^+^ is unique to the GI tract, with the CD103^+^SIRPα^+^ (CD103^+^/signal regulatory protein α^+^) DC subset representing their human counterpart.^9^ CD103^+^ DC as a whole are regulatory, but it is unclear whether specific subsets perform distinct functions because CD103^+^CD11b^+^ DC can control immune tolerance (via retinaldehyde dehydrogenase type 2 (RALDH2) and retinoic acid production),^9^, ^10^, ^11^, ^12^, ^13^ but can also induce helper T-cell 17 (T_H_17) responses;^14^ this latter property suggests some bystander effect and/or redundancy between subsets. CD103^−^ DC can also generate retinoic acid and migrate to lymph nodes.^15^ Although murine studies further our understanding of GI tract DC immunology, they may not always be relevant to humans;^16^, ^17^ for example, RALDH2 activity is not restricted to DC in the human colon but is also expressed in macrophages.^18^

Discrepancies between human and murine DC may reflect environmental and tissue differences as most murine studies focus on the small bowel and human studies are usually of the lower GI tract. There is therefore a need to study human intestinal DC in the context of different anatomic locations as murine DC subsets and function vary throughout the GI tract.^10^, ^19^, ^20^ In agreement with this notion, we have recently showed that human large (colon) and small bowel (terminal ileum) DC have different properties, with a lower proportion of DC producing proinflammatory cytokines in the human colon compared with the (paired) ileum, coupled with a higher capacity of colonic DC to generate Treg cells.^21^ Moreover, colonic and ileal DC have differential abilities to imprint homing properties on the T cells they stimulate, suggesting DC compartmentalization through the human GI tract.^21^ These regional differences in human GI tract DC are in agreement with other observations,^9^ which together suggest the presence of regional immune specialization in the GI tract.^22^

To further explore the possibility that DC change through the GI tract we focused on the human colon, which is structured into different areas. After the terminal ileum (small bowel), the colon starts at the cecum, followed by the right or ascending colon, transverse colon, descending colon, sigmoid colon, and finally the rectum. The right (ascending or proximal side) and left (descending or distal side) part of the human colon have different physiology, embryologic origins, gene expression and epigenetic profiles, microbiota load, and microbiota metabolic activities.^23^, ^24^, ^25^, ^26^ Moreover, proximal and distal colorectal cancers are recognized as different entities, with proximal colorectal cancer having a lower prevalence but a worse prognosis.^27^ Therefore, DC properties may not only be different between the small and the large bowel,^21^ but also between different areas of the colon. Our hypothesis is that in DC subsets, their phenotype and function change through the human GI tract and that DC from different colonic compartments have different immunologic properties.

## Material and Methods

### Biological Samples

Colonic biopsies were obtained at colonoscopy from a total of 95 healthy controls (44.9% males; 57.9 ± 12.9 years (mean ± standard deviation); age interval 28–81). All samples were obtained after bowel preparation for colonoscopy, which can have an impact on mucosa-associated microbiota composition.^28^, ^29^ Samples were obtained in all cases after informed consent and ethics approval from the Hospital Clínico Universitario de Valladolid (Spain) or the Outer West London Research Ethics Committee (United Kingdom). The patients had been referred for colorectal cancer screening or for rectal bleeding or changes in bowel transit. In all cases they had macroscopically and histologically normal intestines. Proximal colon refers to the right or ascending colon (closer to the small intestine); distal colon refers to the left or descending colon (closer to the rectum). Paired biopsies from both the distal and proximal colon were collected (maximum of five biopsies from each area) in ice-chilled complete medium (Dutch modified RPMI 1640 (Sigma-Aldrich, Dorset, United Kingdom) containing 100 μg/mL penicillin/streptomycin, 2 mM l-glutamine, 50 μg/mL gentamicin (Sigma-Aldrich), and 10% fetal calf serum (TCS Cellworks, Buckingham, United Kingdom)), and they were processed within 1 hour. Gender did not affect the expression of any of the studied parameters. Age, however, gave a positive correlation in the proximal colon with mRNA expression of e-cadherin (Pearson’s *r*: 0.66, *P* = .035) and microbiota load as determined by quantifying 16S rRNA (Pearson’s *r*: −0.75, *P* = .011).

Human peripheral blood was collected from healthy volunteers with no known autoimmune or inflammatory diseases, allergies, or malignancies after informed consent had been obtained. Peripheral blood mononuclear cells (PBMC) were obtained by centrifugation over Ficoll-Paque PLUS (Amersham Biosciences, Chalfont St. Giles, United Kingdom). Some PBMC were washed twice in phosphate-buffered saline containing 1 mM EDTA and 0.02% sodium azide (fluorescence-activated cell sorting [FACS] buffer) and stained with fluorochrome-conjugated antibodies; others were used to enrich naive T cells.

### Lamina Propria Dendritic Cells

**Figure 1:**
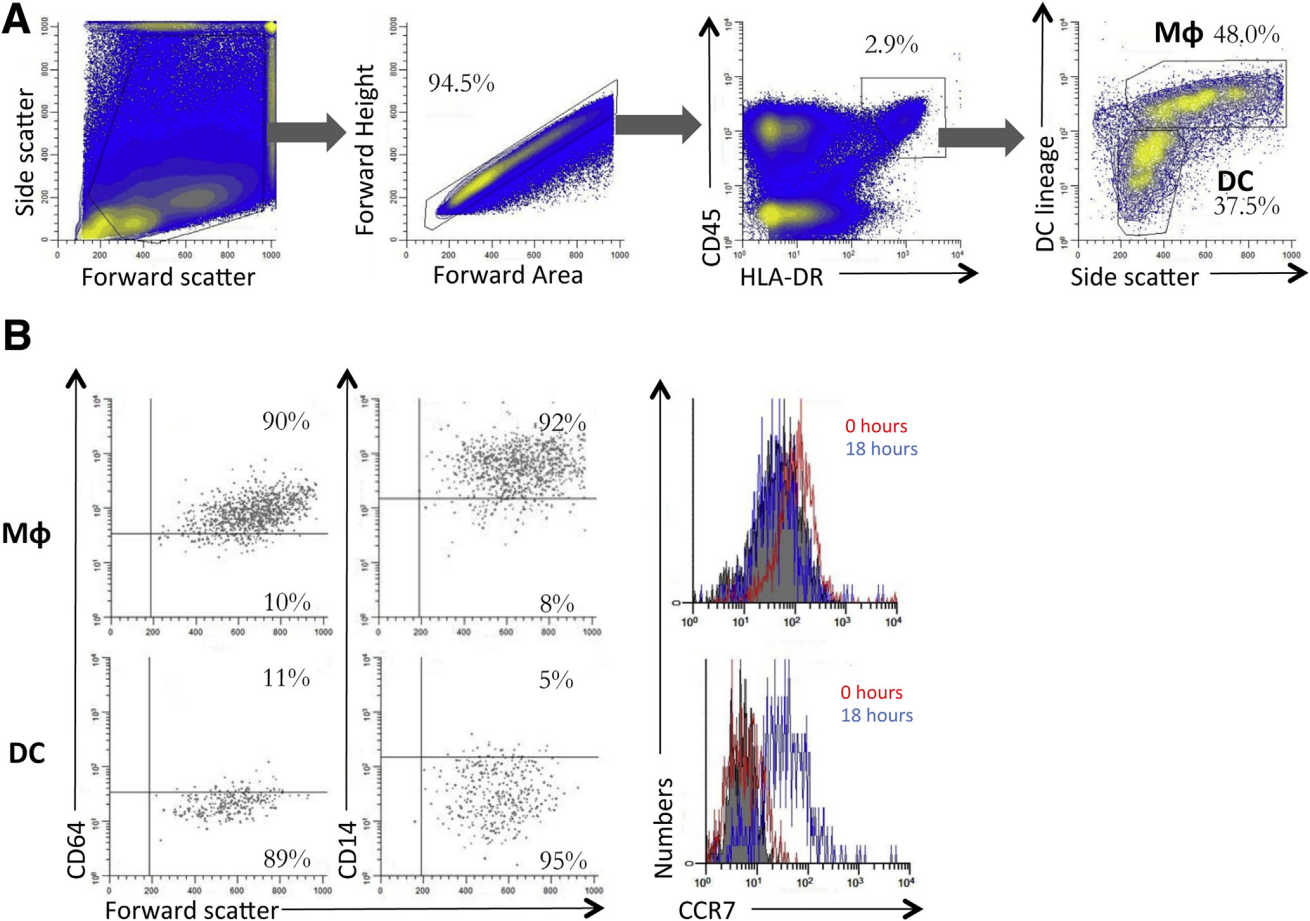

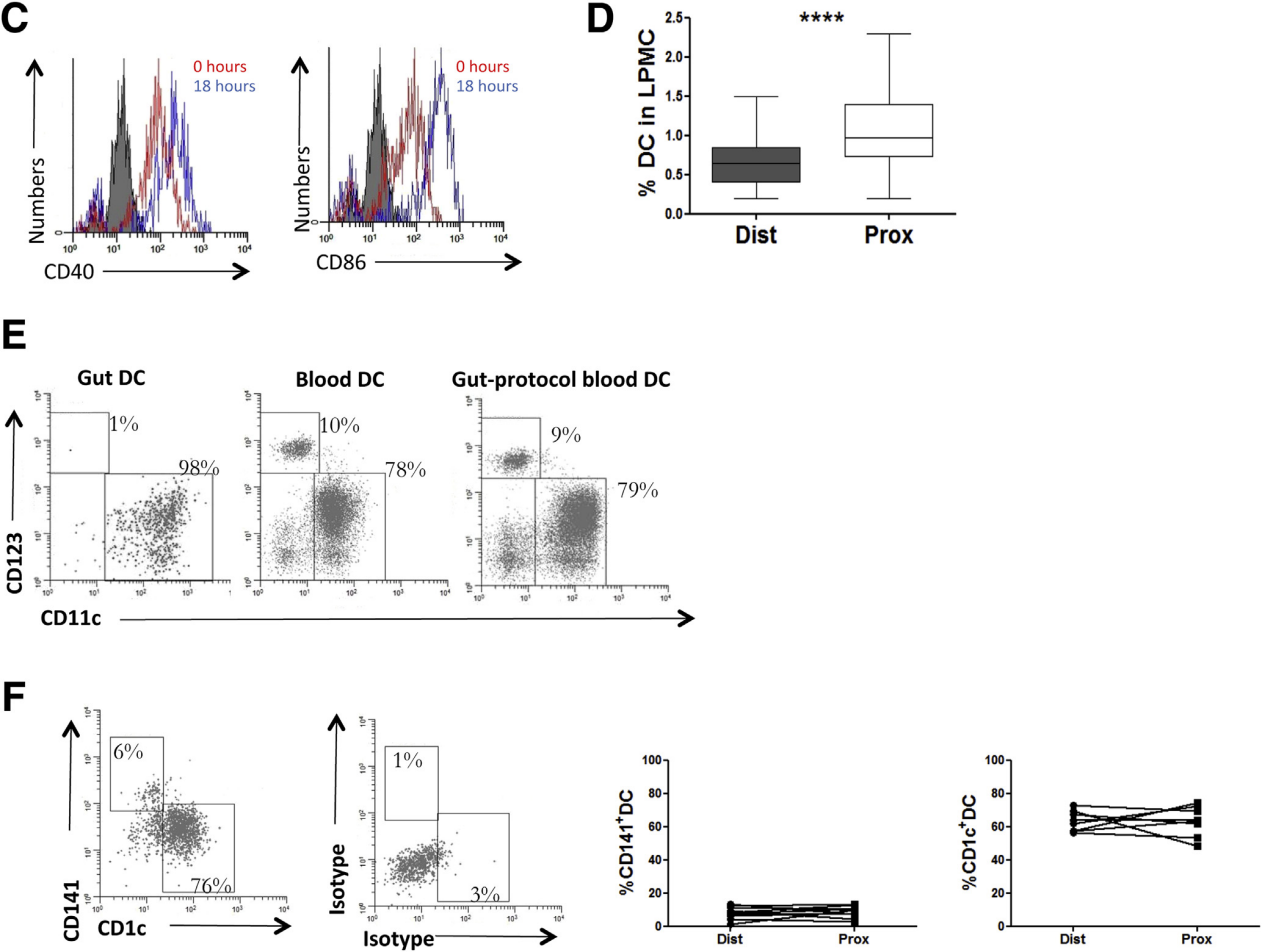
**Identification of human colonic dendritic cells (DC).** (*A*) Total antigen-presenting cells (CD45^+^HLA-DR^high^) were identified within single viable cells (based on the forward/side scatter properties). DC were further discriminated from macrophages (Mφ) based on lineage marker expression (CD3, CD14, CD16, CD19, CD34). (*B*) In contrast to putative macrophages (CD45^+^HLA-DR^+^lineage^+^), DC (CD45^+^HLA-DR^+^lineage^−^) were CD64^−^CD14^−^ and up-regulated CCR7 expression after overnight culture (blue histogram) as well as (*C*) CD40 and CD86, compared with fresh noncultured cells (red histogram). Shaded histogram in *B* and *C* denotes isotypes. (*D*) Percentages of DC within the total number of single lamina propria mononuclear cells (LPMC) were higher in the proximal (Prox) colon compared with the distal (Dist) (n = 43). (*E*) Lamina propria DC were mainly CD11c^+^ myeloid DC (mDC). Lack of non-mDC in the human colon was unlikely to be due to sample processing, as distribution of CD11c^+^ and CD123^+^ blood DC was not altered after dithiothreitol + EDTA + collagenase incubation (gut-protocol) of peripheral blood mononuclear cells. (*F*) Most human colonic DC were CD1c^+^, in contrast to CD141^+^DC, with no differences between the proximal and distal colon. Data shown in *B*, *C*, and *E* are representative of two independent experiments. Paired *t* tests were applied in *D* and *F. P* < .05 was considered statistically significant (****P < .0001).

Lamina propria DC were characterized by flow cytometry as previously described elsewhere.^21^, ^30^, ^31^, ^32^ Briefly, freshly obtained biopsies were incubated with Hank’s balanced salt solution (GIBCO BRL, Paisley, Scotland, United Kingdom) containing 1 mM dithiothreitol for 20 minutes and then in 1 mM EDTA solutions to remove the associated mucus/bacteria and epithelial layer, respectively. Lamina propria mononuclear cells (LPMC) were obtained from biopsy tissue after quick collagenase digestion with 1 mg/mL of collagenase D (Roche Diagnostics, Lewes, United Kingdom). LPMC were collected by centrifugation, washed twice in FACS buffer, and stained with fluorochrome-conjugated antibodies. Viable DC were identified by flow cytometry within single live cells as CD45^+^ HLA-DR^+^ lineage (CD3 CD14 CD16 CD19 CD34)^−^ based on Forward and Side scatter characteristics (Figure 1*A*).

In some cases, LPMC were cultured overnight (37°C, 5% CO_2_, high humidity) and subsequently enriched for DC after density-gradient centrifugation (600*g*, 15 minutes at room temperature) using NycoPrep (Progen Biotechnik, Heidelberg, Germany), and were then cultured with T cells. The cells collected from the interface (typically 25,000–50,000 cells) have been characterized as functional colonic DC.^17^, ^21^, ^30^, ^31^, ^32^, ^33^, ^34^

### Antibody Labeling

LPMC and/or PBMC were stained with monoclonal antibodies for DC characterization. Supplementary Table 1 shows the specificity, clone fluorochrome, and sources of the antibodies used. Cells were labeled in FACS buffer. Labeling was performed on ice and in the dark for 20 minutes. Cells were washed twice in FACS buffer, fixed with 1% paraformaldehyde in 0.85% saline, and stored at 4°C before acquisition on the flow cytometer (within 48 hours). Appropriate isotype-matched control antibodies were purchased from the same suppliers. For intracellular staining, cells were fixed with Leucoperm A (AbD Serotec, Kidlington, United Kingdom) after surface staining and permeabilized with Leucoperm B (AbD Serotec) before intracellular staining.

Endocytic activity of DCs was determined by uptake of fluorescein isothiocyanate (FITC)-dextran (molecular mass 40 kDa) (100 μg/mL; Sigma-Aldrich), 30 minutes at 37°C or on ice (internal control).^21^, ^33^ The surface staining for flow cytometry acquisition was subsequently performed as described here.

### Proliferation Assays

CD4^+^ naïve T-cells were obtained from freshly isolated PBMC, suspended in MiniMACs buffer (phosphate-buffered saline containing 0.5% bovine serum albumin and 2 mM EDTA) and depleted of CD14, CD19, CD8, CD45RO, and HLA-DR positive cells with immunomagnetic beads (Miltenyi Biotech, Bisley, United Kingdom) following the manufacturer’s instructions. CD4^+^ naive T-cells were labeled with 10 μM 5-carboxy fluorescein diacetate succinimidyl ester (CFSE) (Invitrogen/Life Technologies, Paisley, United Kingdom) and cultured (4 × 10^5^/well) for 5 days in *U*-bottomed 96-well microtiter plates (BD Biosciences, Oxford, United Kingdom) with 0, 1%, 2%, or 3% enriched allogeneic colonic DC. Cells were recovered, and CFSE^low^ proliferating cells were identified, quantified, and phenotyped by flow cytometry.

### Culture Supernatants

Freshly obtained intestinal biopsy samples were immediately cultured in complete medium (1 biopsy per 1 mL of complete medium per well) for 24 hours, after which culture media were filtered (0.45 μm) and cell-free supernatants preserved (−80°C) until they were analyzed using a FlowCytomix Multiple Analyte Detection (eBioscience, Wien, Austria) on a BD CantoII flow cytometer (BD Biosciences), following the manufacturer’s instructions, for the concentration of interferon (IFN) γ (detection limit [DL]: 0.25 pg/mL), IFNα (DL: 4.04 pg/mL), interleukin-1β (IL-1β) (DL: 1.80 pg/mL), IL-2 (DL: 0.35 pg/mL), IL-4 (DL: 1.22 pg/mL), IL-5 (DL: 0.76 pg/mL), IL-6 (DL: 4.10 pg/mL), IL-7 (DL: 3.18 pg/mL), IL-9 (DL: 1.17 pg/mL), IL-10 (DL: 2.88 pg/mL), IL-12p70 (DL: pg/mL), IL-13 (DL: 0.43 pg/mL), IL-17A (DL: 0.89 pg/mL), IL-22 (DL: 3.98 pg/mL), IL-23 (DL: 29.14 pg/mL), IL-27 (DL: 0.79 pg/mL), leptin (DL: 34.95 pg/mL), and tumour necrosis factor-α (DL: 3.10 pg/mL). IgA content was determined via radial immunodiffusion kit (DL: 8.5 mg/L; Kit IgA RID-ML, Binding Site Group, Birmingham, United Kingdom) following the manufacturer’s instructions. Values below the level of detection were reported as equal to it.

### Quantitative Reverse-Transcription Polymerase Chain Reaction

Freshly obtained intestinal biopsies were immediately embedded in RNAlater (Ambion/Life Technologies, Paisley, United Kingdom) and snap-frozen (−80°C). Total RNA was isolated from each biopsy using the TRIzol reagent according to the protocol provided by the manufacturer. Reverse transcription was performed by using the SuperScript First-Strand Synthesis System for Reverse-Transcriptase-PCR Kit (Invitrogen/Life Technologies) using random hexamers. The mRNA levels of RALDH2, CCL25, MADCAM1, e-cadherin, and GADPH (housekeeping gene) were measured by real-time polymerase chain reaction (PCR) by using a LightCycler instrument (Roche Applied Science, Mannheim, Germany). Microbiota load was determined via 16S rRNA amplification. Reactions were performed using the FastStart SYBR Green MasterMix (Roche Diagnostics) with thermolabile uracil DNA glycosylase (Roche Diagnostics) to prevent carryover contamination. Primer sets and PCR conditions are described in Supplementary Table 2. Levels are expressed as the ratio molecule/GADPH in arbitrary units (U).

### Migration Experiments

We placed 1 million fresh PBMC in the upper insert of transwell culture plates, and their capacity to migrate through 3 μm pores was assessed by flow cytometry after collection of the cells in the lower chamber. Colonic biopsy tissues from the proximal and distal colon were cultured as above in 24-well culture plates (three biopsy samples per well in 1.5 mL of complete medium) for 18 hours, after which the samples were filtered (0.45 μm) and the cell-free culture supernatants used immediately in the lower chamber of transwell culture plates (Sigma-Aldrich).

In some experiments, the colonic supernatants were incubated (20 minutes, room temperature) with anti-chemokine (C-C motif) ligand 2 (anti-CCL2, 1 μg/mL) before performing the migration experiments. In other cases, the lower chamber contained fresh complete medium supplemented with different doses of human recombinant CCL2. The addition of fluorescent counting beads (Flow-count Fluorospheres; Beckman Coulter, Brea, CA) before flow cytometry acquisition allowed determination of the absolute number of migrated cells into the lower chamber. The results are shown as a ratio compared with the basal or spontaneous migration.

### Flow Cytometry and Data Analysis

Cells were acquired on a BD Canto II (BD Biosciences) or a LSR Fortessa flow cytometer (BD Biosciences) and analyzed using WinList 3D 7.1 software (Verity Software House, Topsham, ME). The proportion of cells positive for a given marker was determined by reference to staining with an isotype-matched control antibody.

### Electron Microscopy

Electron microscopy characterization of colonic DC was performed as previously described elsewhere.^21^, ^33^, ^35^ Briefly, between 200,000 and 1,000,000 LPMC were fixed in 3% glutaraldehyde in 0.1 M sodium phosphate buffer (SPB), pH 7.4, for up to 2 hours at room temperature and then were maintained at 4°C until they were processed. The cells were washed in the 0.1 M SPB (pH 7.4) and embedded as a pellet in low-gelling-temperature agarose (Sigma-Aldrich). They were washed in 0.1 M SPB (pH 7.4) and postfixed in 1% osmium tetroxide in 0.1% SPB (pH 7.4) for 1 hour. The samples were then washed and left in distilled water overnight to remove the phosphate and were block stained in 2% uranyl acetate for 2 to 4 hours. The samples were washed with dH_2_O and dehydrated using an acetone gradient and gradually infiltrated with Araldite resin. The Araldite was changed twice over 4 to 8 hours, and the samples were embedded in the Araldite resin and then cured for 18 hours at 65°C.

Ultrathin sections (100 nm thick) were cut on a Reichert-Jung Ultracut E microtome (Leica, Wetzlar, Germany) and collected on a 200 mesh copper grid. After staining with Reynold’s lead citrate, the grid was carbon coated and visualized using a JEOL 1200 EX electron microscope (JEOL, Tokyo, Japan).

The DC were identified as previously described elsewhere.^33^, ^34^, ^35^, ^36^ The DC were subsequently classified into mature and immature DC based on their morphology by electron microscopy. Immature DC were small cells with short veils relative to their size, a small area of cytoplasm, and heterochromatic nuclei with thick chromatin layer surrounding the nucleus and chromatic dense clusters within the nucleus. In contrast, mature DC had long veiled projections.^21^

### Microbiota Analysis

Paired biopsy samples from the proximal and distal colon were collected from a total of 19 healthy controls (9 women, 10 men; mean age: 59.7 ± 10.2 years) with no known autoimmune disease and/or malignancies. The biopsy tissues were immediately embedded in RNAlater (Ambion) and snap-frozen at −80°C. DNA was extracted using the FastDNA SPIN Kit for Soil in conjunction with a Fastprep-24 machine (MP Biomedicals, Santa Ana, CA) following the manufacturer’s protocols.

The V1–V2 regions of bacterial 16S rRNA genes were PCR amplified using Q5 Taq polymerase (New England BioLabs, Ipswich, MA) and Golay barcoded primers (purchased from Eurofins MWG Operon, Huntsville, AL) MiSeq-27F (5′-AATGATACGGCGACCACCGAGATCTACACTATGGTAATT CCAGMGTTYGATYMTGGCTCAG-3′) and MiSeq-338R (5′-CAAGCAGAAGACGGCATACGAGAT-barcode-AGTCAGTCAGAAGCTGCCTCCCGTAGGAGT-3′). Each sample included in the study was amplified with a 338R primer containing a unique 12-mer Golay barcode sequence. The PCR conditions were as follows: 98°C for 2 minutes followed by 25 cycles of 98°C for 30 seconds, 50°C for 30 seconds, 72°C for 1 minute and 30 seconds, and then a final extension step at 72°C for 5 minutes. Four PCR reactions per sample were performed, which were then pooled into a single amplicon mix for each of the samples. The PCR amplicons from each sample were then quantified using a Qubit 2.0 Fluorometer (Invitrogen/Life Technologies), and equimolar concentrations of each sample were added together into a final mastermix for sequencing on an Illumina MiSeq machine (2 × 250 bp read length) (Illumina, San Diego, CA).

Sequence data have been deposited in the European Nucleotide Archive (http://www.ebi.ac.uk/ena) under study number ERP007146. The individual accession numbers for each of the biopsy samples are shown in Supplementary Table 3.

The resulting sequence data was processed using the mothur software package (http://www.mothur.org/), closely following their MiSeq SOP.^37^ In brief, paired contigs were created from the forward and reverse MiSeq reads, and all contigs shorter than 260 bp (base pairs) and longer than 450 bp, and those containing any ambiguous bases or homopolymeric stretches of longer than seven bases were removed. Chimeras were removed using Perseus^38^ as implemented in mothur. Operational taxonomic units (OTUs) were generated from preclustered data (diffs = 3) at the 97% cutoff level and then assigned a taxonomic classification using the Ribosomal Database Project (RDP) taxonomy provided with mothur. Diversity measures such as Shannon and inverse Simpson indices were calculated in mothur, where every sample was first subsampled down to 450 reads to ensure equal sequencing depth for these comparisons. Good’s coverage (an estimate of completeness of species sampling) at this sequencing depth was on average 93.4%. A cluster dendrogram using the Bray Curtis calculator was generated in mothur and visualized using the iTOL online resource (http://itol.embl.de/).^39^ Differences in bacterial community structure between proximal and distal colonic biopsies were assessed using the analysis of molecular variance (AMOVA), parsimony, Metastats,^40^ and LEfSe^41^ tests as implemented in the mothur software package.

### Statistical Analyses

Two-tailed paired *t* test, two-tailed Pearson's correlation, and two-way paired analysis of variance (ANOVA) were applied as explained in each section/figure legend. In the case of multiple comparisons, subsequent ad hoc Bonferroni correction was applied. Microbiota data were compared using the AMOVA, parsimony, Metastats,^40^ and LEfSe^41^ tests as implemented in the mothur software package.^42^ *P* < .05 was considered statistically significant.

## Results

### Identification of Human Colonic Dendritic Cells

Antigen-presenting cells (CD45^+^HLA-DR^high^) were identified within LPMC. DC discrimination from macrophages (Mφ) and B cells was based on lack of expression of markers of other lineages (Figure 1*A*), as we and others have previously described.^17^, ^18^, ^21^, ^31^, ^32^, ^34^ DC also had lower granularity as they lack Mφ vacuolar systems, aiding discrimination between DC and Mφ. Further analysis confirmed that DC gated in this way were CD14^−^ and CD64^−^ (see Figure 1*B*). Additionally, in contrast to Mφ, DC up-regulated chemokine (C-C motif) receptor 7 (CCR7) expression after 18 hours of culture (Figure 1*B*).^18^ Culture also induced DC maturation (see Figure 1*C*), and these DC were used in functional experiments for T-cell stimulatory capacity,^21^, ^31^, ^33^ avoiding the need for additional exogenous stimulation.

The DC numbers were statistically significantly higher in the proximal colon (see Figure 1*D*), with all DC in both compartments being mDC (CD11c^+^CD123^-^) (see Figure 1*E*). The inability to detect non-mDC in human colonic biopsies was not due to sample processing and/or collagenase digestion as this protocol was able to detect plasmacytoid dendritic cells (pDC) in PBMC samples processed in parallel with colonic biopsy tissues (see Figure 1*E*). This is in agreement with recent observations suggesting that non-mDC in the human GI tract are scarce or absent.^18^, ^43^ Further analysis of colonic mDC confirmed that most were CD1c^+^ DC and not CD141^+^ DC (see Figure 1*F*), with no differences between compartments.

### Dendritic Cell Subsets in the Human Colon

**Figure 2:**
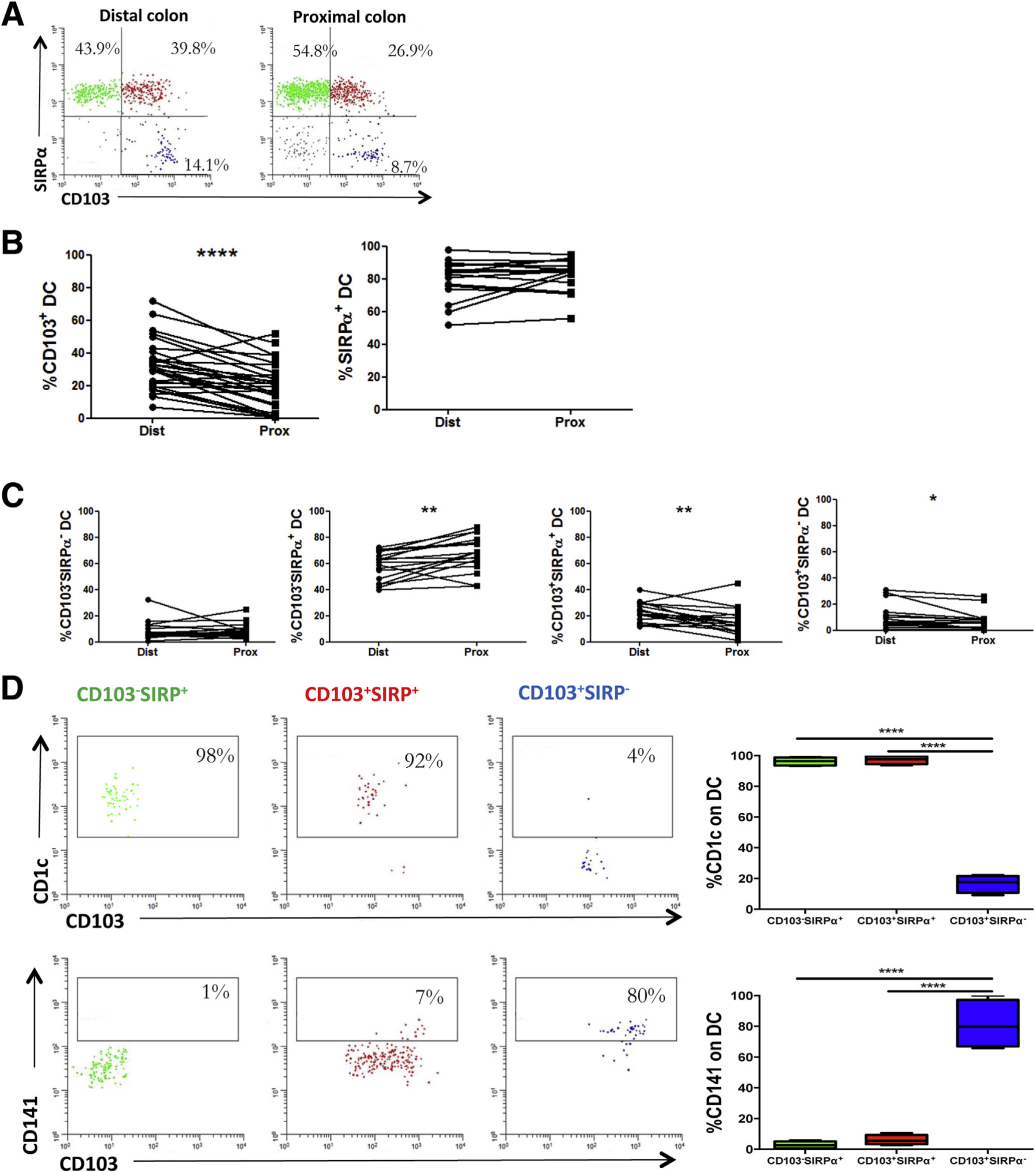

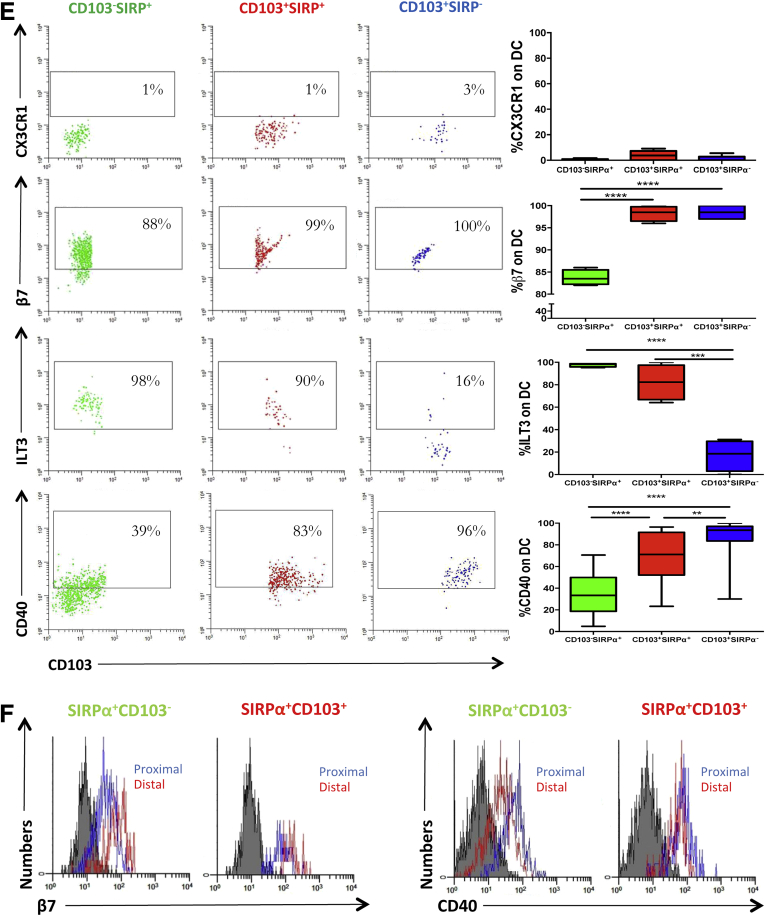
**Dendritic cell (DC) subsets in the human colon.** (*A*) DC were identified (as in Figure 1*A*) and were divided into subsets based on expression of CD103 and SIRPα. (*B*, *C*) Relative proportions of DC in subsets in the proximal (Prox) and distal (Dist) human colon. (*D*) CD103^−^SIRPα^+^, CD103^+^SIRPα^+^, and CD103^+^SIRPα^−^ DC were characterized for CD1c and CD141; and also for (*E*) CX3CR1, ILT3, CD40, and β7. (*F*) SIRPα^+^CD103^−^ and SIRPα^+^CD103^+^ DC subsets were identified (as in *A*), and β7 and CD40 expression was studied in biopsy samples from the proximal (*blue*) and distal (*red*) human colon compared with the isotype (shaded histogram). Results from *D* and *E* are from at least five independent experiments; results from *F* are representative of three independent experiments. Paired *t* test (*B* and *C*) and paired one-way analysis of variance with Bonferroni’s ad-hoc analysis (*D*, *E*) were applied. *P* < .05 was considered statistically significant (**P < .01; ***P < .001; ****P < .0001).

Comparisons of DC subsets derived from the proximal and distal human colon were made based on CD103 and SIRPα expression^9^, ^21^ (Figure 2*A*). SIRPα^+^ DC were equally distributed in each compartment, but there was a lower proportion of CD103^+^ DC in the proximal colon biopsy samples (see Figure 2*B*), which translated into lower numbers of CD103^+^SIRPα^+^ DC (analogous to murine CD103^+^CD11b^+^ DC)^9^ at this site (see Figure 2*C*). Further characterization revealed that SIRPα^+^ DC (both CD103^+^ and CD103^−^) were typically CD1c^+^ whereas CD103^+^SIRPα^−^ were CD141^+^ DC (see Figure 2*D*).^9^, ^10^ CX3CR1 was virtually absent in all colonic DC (see Figure 2*E*) but was expressed by macrophages,^17^ whereas the GI-tract homing β7 integrin was expressed by the majority of colonic DC, with all CD103^+^ DC being positive. SIRPα, which regulates homeostatic properties of intestinal DC,^44^ was typically coexpressed with the inhibitory receptor Ig-like transcript 3 (ILT3),^45^ with no differences between CD103^-^SIRPα^+^ and CD103^+^SIRPα^+^ DC; CD103^+^SIRPα^−^ DC were ILT3^−^. Conversely, ILT3^−^ DC displayed a higher expression of the costimulatory molecule CD40 (see Figure 2*E*).

Having characterized human colonic DC subsets, we next determined whether proximal and distal human colonic DC subsets had differences in activation/maturation and homing marker expression. To this end, we examined expression of CD40 (representative of DC maturation status) and β7 (indicative of DC gut-homing capacity) on different DC subsets. Given the low numbers of DC obtained from human colonic biopsy samples (see Figure 1*D*), and that SIRPα^−^ cells were scarce in all locations (see Figures 2*A*–*C*), these markers were studied in CD103^+^SIRPα^+^ and CD103^-^SIRPα^+^ DC subsets (analogous to murine CD103^+^CD11b^+^ and CD103^+^CD11b^−^ subsets, respectively)^9^ to ensure measurable effects. Both subsets from the proximal colon revealed lower β7 and higher CD40 expression compared with their distal counterparts (see Figure 2*F*), suggesting that alterations in DC exist between the proximal and distal compartments of human colon irrespective of the DC subset.

### Chemokine (C-C Motif) Receptor 2 Mediates Dendritic Cell Recruitment in the Colon

**Figure 3:**
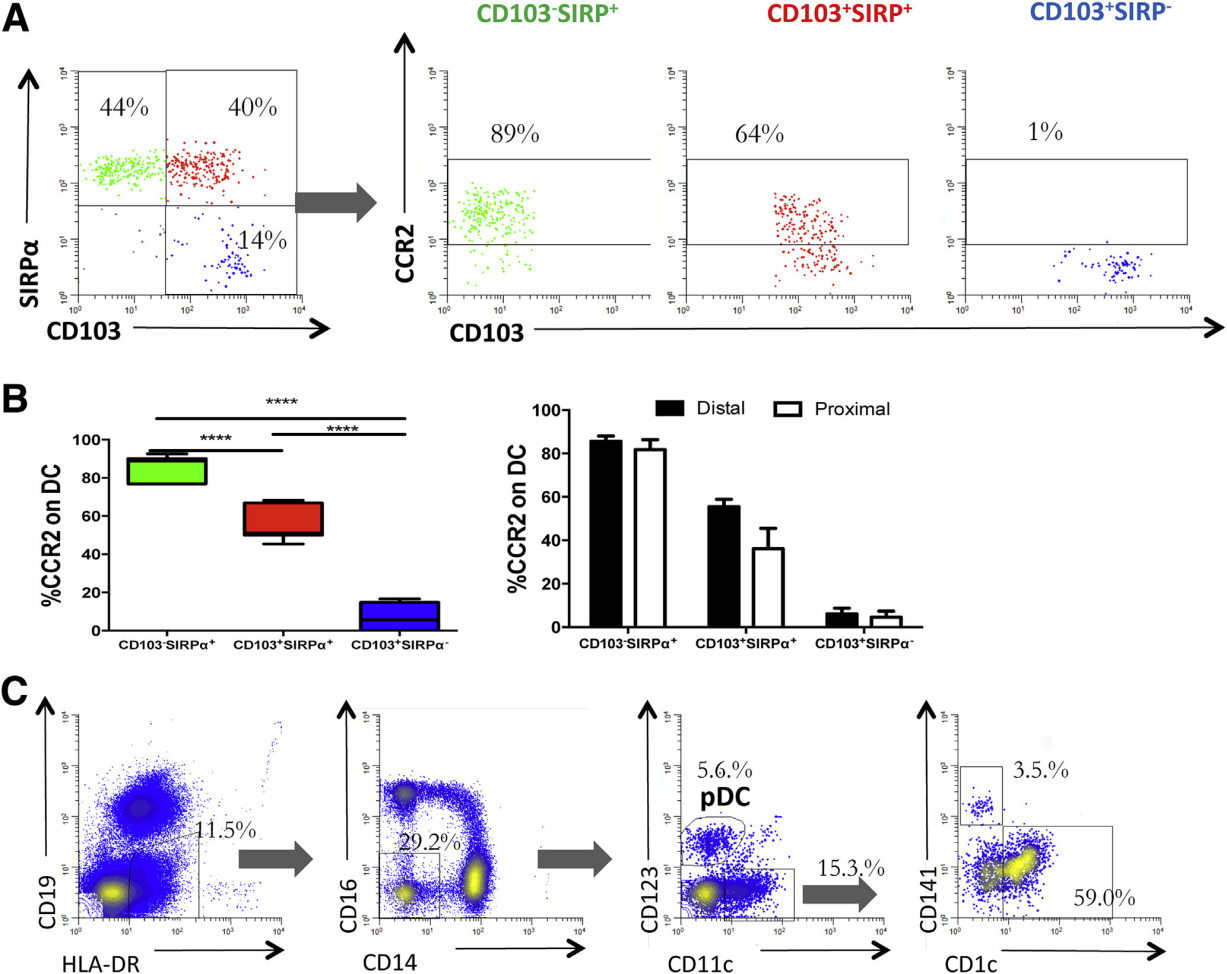

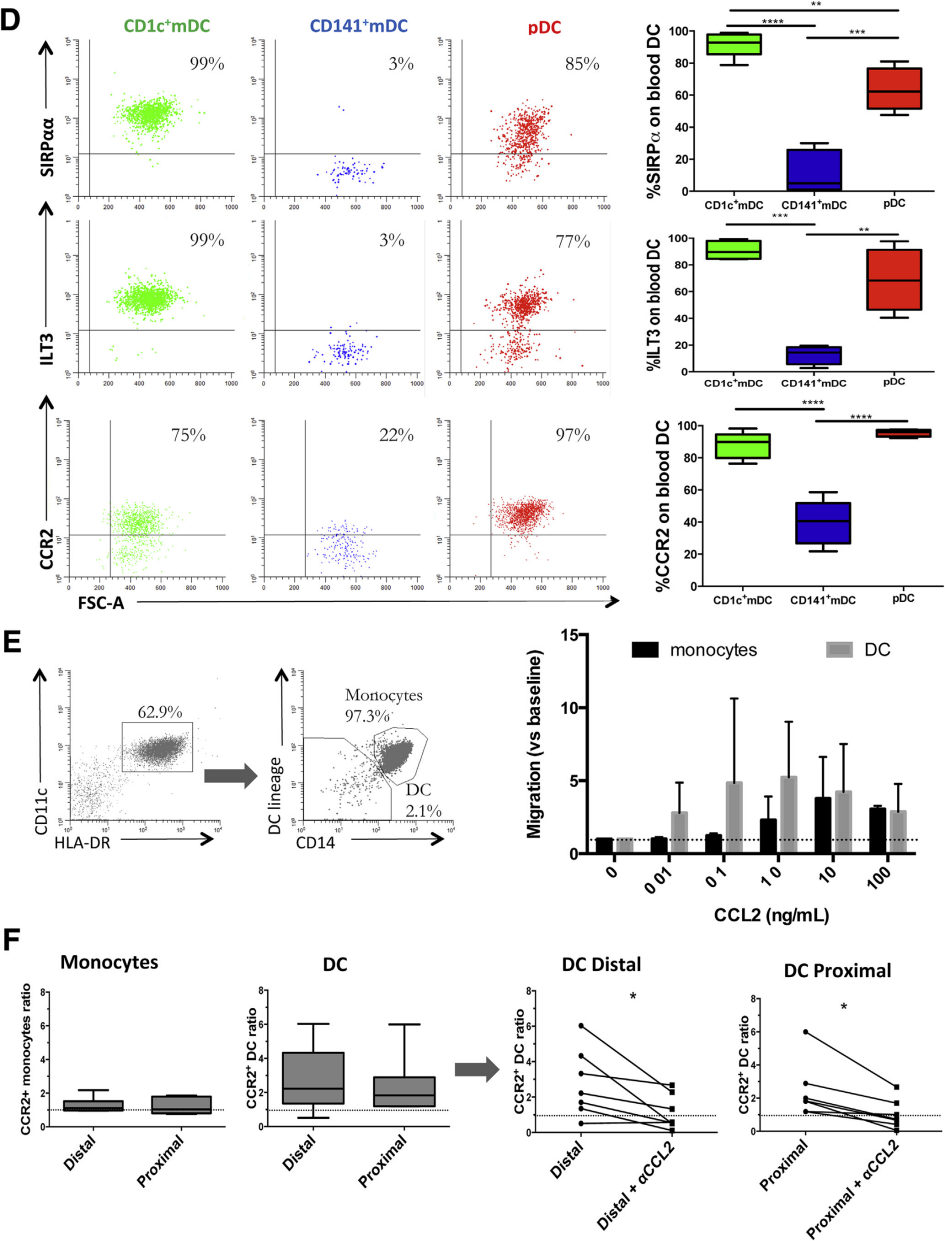
**Chemokine (C-C motif) receptor 2 (CCR2) mediates blood dendritic recruitment by the human colon.** (*A*) CD103^−^SIRPα^+^, CD103^+^SIRPα^+^, and CD103^+^SIRPα^−^ dendritic cells (DC) were identified (as in Figure 2*A*) and assessed for the expression of CCR2. (*B*) Pooled data of CCR2 expression on different subsets and in the proximal and distal colon. (*C*) Peripheral blood mononuclear cells (PBMC) studied by flow cytometry. After exclusion of CD19, CD14, and CD16, DC were identified within HLA-DR^+^ as plasmacytoid (pDC, CD123^+^) or myeloid (mDC, CD11c^+^). The mDC were further divided into CD1c^+^ and CD141^+^. (*D*) Expression of SIRPα, ILT3, and CCR2 was determined on peripheral CD1c^+^ mDC, and CD141^+^ mDC and pDC. (*E*) After 1 million PBMC were placed in the upper insert of a transwell chamber, their migration toward different concentrations of CCL2 was determined 3 hours later. Total migrated CD11c^+^HLA-DR^+^ cells were identified by flow cytometry and further divided into monocytes (CD14^+^lineage^+^) and DC (CD14^−^lineage^−^). (*F*) Migration of CCR2^+^ monocytes and DC toward cell-free biopsy culture supernatants from the proximal and distal human colon. DC migration was also determined with and without specific blockage of anti-CCL2 in the culture supernatants. Results from the transwell experiments (*E*, *F*) are displayed as the ratio of migrated cells compared with the basal migration (*dotted line*). Results from *B* and *D* are from at least five independent experiments. Results from *E* summarize between three and six independent experiments per condition; results from F summarize seven independent experiments. Paired *t* tests were applied, and *P* < .05 was considered statistically significant (*P < .05; **P < .01; ***P < .001; ****P < .0001).

We next studied the potential mechanisms that may account for blood DC recruitment by the human colon to see whether there was a differential recruitment capacity elicited by the proximal and distal sides. CCR2 mediates monocyte recruitment in the intestinal mucosa, although its expression may not be restricted to monocytes/macrophages as Scott et al^20^ have identified a novel CCR2^+^ DC subset within murine and human intestinal DC. CCR2 was expressed by most colonic CD103^−^SIRPα^+^DC with expression being variable on CD103^+^SIRPα^+^ and absent on CD103^+^SIRPα^−^ DC (Figure 3*A*), with no differences between the proximal and distal side of the human colon (see Figure 3*B*).

CCR2^+^ APC numbers are increased in the coeliac duodenum after in vivo gluten challenge,^46^ suggesting intestinal recruitment of blood precursors. We therefore characterized CCR2 expression on human circulating DC. Blood DC were identified within HLA-DR^+^ cells (after exclusion of CD14^+^CD16^+^CD19^+^ cells) and divided into subsets based on the expression of CD123 (pDC), CD11c/CD1c (CD1c^+^ mDC), and CD11c/CD141 (CD141^+^ mDC)^5^ (see Figure 3*C*). Circulating CD1c^+^ mDC were SIRPα^+^ILT3^+^CCR2^+^ in contrast to circulating CD141^+^ mDC, which were SIRPα^-^ILT3^-^CCR2^−^ (see Figure 3*D*). Circulating CD1c^+^ mDC therefore displayed a phenotype similar to intestinal SIRPα^+^ DC (CD1c^+^ILT3^+^CCR2^+^) whereas circulating CD141^+^ mDC had a phenotype similar to intestinal CD103^+^SIRPα^−^ DC (CD141c^+^ILT3^-^CCR2^−^) (see Figures 2 and 3). These results are in agreement with recent observations relating intestinal SIRPα^+^ DC to circulating CD1c^+^ mDC and intestinal CD103^+^SIRPα^−^ DC to circulating CD141^+^ mDC.^9^

The similar phenotype of intestinal SIRPα^+^ DC and circulating CD1c^+^ mDC, and the expression of CCR2 on both DC subsets suggest that they might be recruited to the colonic mucosa in a CCR2-dependent manner. To explore this possibility, we first confirmed that CCR2 on circulating mDC was functional; these cells (together with monocytes) migrated in a dose-dependent manner toward CCL2 in transwell migration assays (see Figure 3*E*). Finally, we studied whether cell-free colonic culture supernatants attracted circulating mDC. Although circulating monocytes were not attracted by the colonic culture supernatants, DC had a higher migration (compared with the basal) toward distal (*P* = .04) and proximal (*P* = .06) cell-free culture supernatants; migration was CCR2 dependent as migration was reduced if the culture supernatants had been previously blocked with anti-CCL2 (see Figure 3*F*). These results confirm that CCR2 expression is functional on circulating mDC and that it mediates blood DC recruitment by the human colon. However, there was no differential capacity of the proximal and distal sides of the colon to recruit circulating DC.

### Proximal Colon Dendritic Cells Are More Mature and Have Lower Homing Marker Expression

Given that there was not a differential capacity to recruit circulating DC by the proximal or distal human colon (see Figure 3), and that DC differences between the proximal and distal compartments occurred in all DC subsets (see Figure 2*F*), we next characterized in more detail the phenotype and function of total mDC in each compartment.

**Figure 4:**
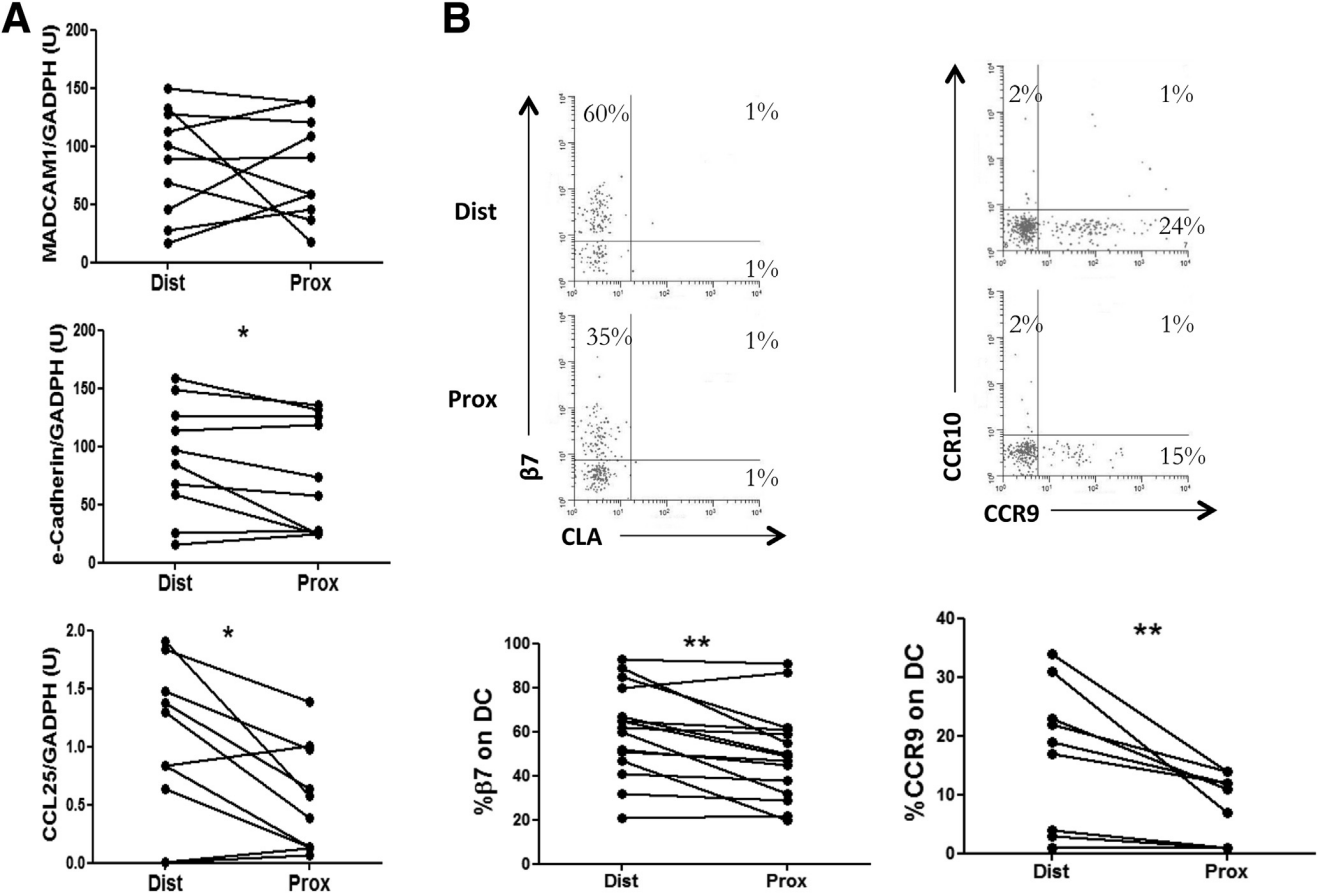

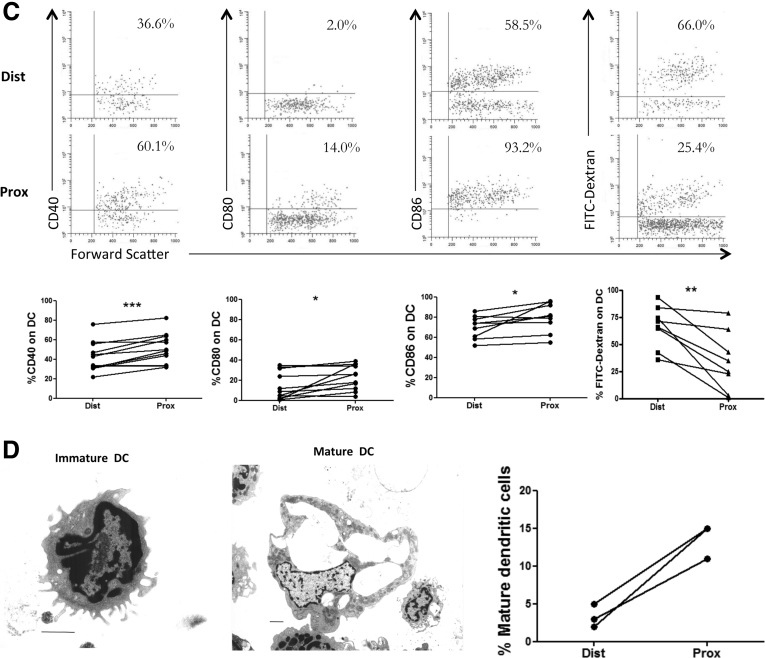
**Proximal colon dendritic cells (DC) have lower homing marker expression and are more mature.** (*A*) The expression of mRNA of the gut-homing ligands MADCAM1, e-cadherin, and CCL25 was determined on noncultured biopsy tissues by quantitative reverse-transcription polymerase chain reaction, with the results displayed in arbitrary units (U). (*B*) DC from the proximal (Prox) and distal (Dist) human colon were identified (as in Figure 1*A*), and the expression of β7, cutaneous lymphocyte antigen (CLA), CCR9, and CCR10 were determined by flow cytometry. (*C*) Expression of costimulatory molecules (CD40, CD80, CD86) and fluorescein isothiocyanate–dextran endocytic capacity was also determined by flow cytometry. (*D*) Lamina propria dendritic cells were identified by electron microscopy in distal (Dist) and proximal (Prox) human colon biopsy samples. DC were characterized as immature and mature according to heterocromatic/euromatic nuclei and veil/dendrite extension. The percentage of mature DC is referred to the total number of studied DC in the proximal and distal colon. For the flow cytometry experiments (*B*, *C*), regions were set according to isotype controls (not shown). Histograms show paired results from several independent experiments. Paired *t* tests were applied, and *P* < .05 was considered statistically significant (*P < .05; **P < .01; ***P < .01).

As human proximal and distal colon DC expressed different levels of β7 (see Figure 2*F*), we further studied the expression and potential function of gut-homing molecules β7 and CCR9^17^, ^31^ by DC isolated from the proximal compared with the distal colon. MadCam1 (α4β7 ligand) expression was equally distributed throughout the proximal and distal colon although both e-cadherin (αEβ7 ligand) and CCL25 (CCR9 ligand) mRNA expression were lower in proximal colon DC (Figure 4*A*). Fitting with the expression patterns of their corresponding ligands, β7 and CCR9 expression were lower among DC from the proximal colon, with the skin- and mucosal-homing cutaneous lymphocyte antigen (CLA) and CCR10 molecules being absent (see Figure 4*B*).

We next determined whether proximal/distal colonic DC had a different maturational status, as suggested by higher CD40 expression displayed by proximal colon DC (see Figure 2*F*). DC in the proximal colon showed higher expression of CD40, CD80, and CD86 and lower endocytic capacity as assessed by uptake of FITC-dextran dye, suggesting greater maturity (see Figure 4*C*). Lamina propria DC were also studied by electron microscopy to further analyze their maturation status as previously reported.^21^, ^35^ There was a higher proportion of mature DC in the proximal colon as identified by their longer veils/dendrites relative to their size and euchromatic nuclei (see Figure 4*D*).^35^

### Proximal Colonic Dendritic Cells Are More Stimulatory With Lower Gut-Homing Imprinting Capacity

**Figure 5:**
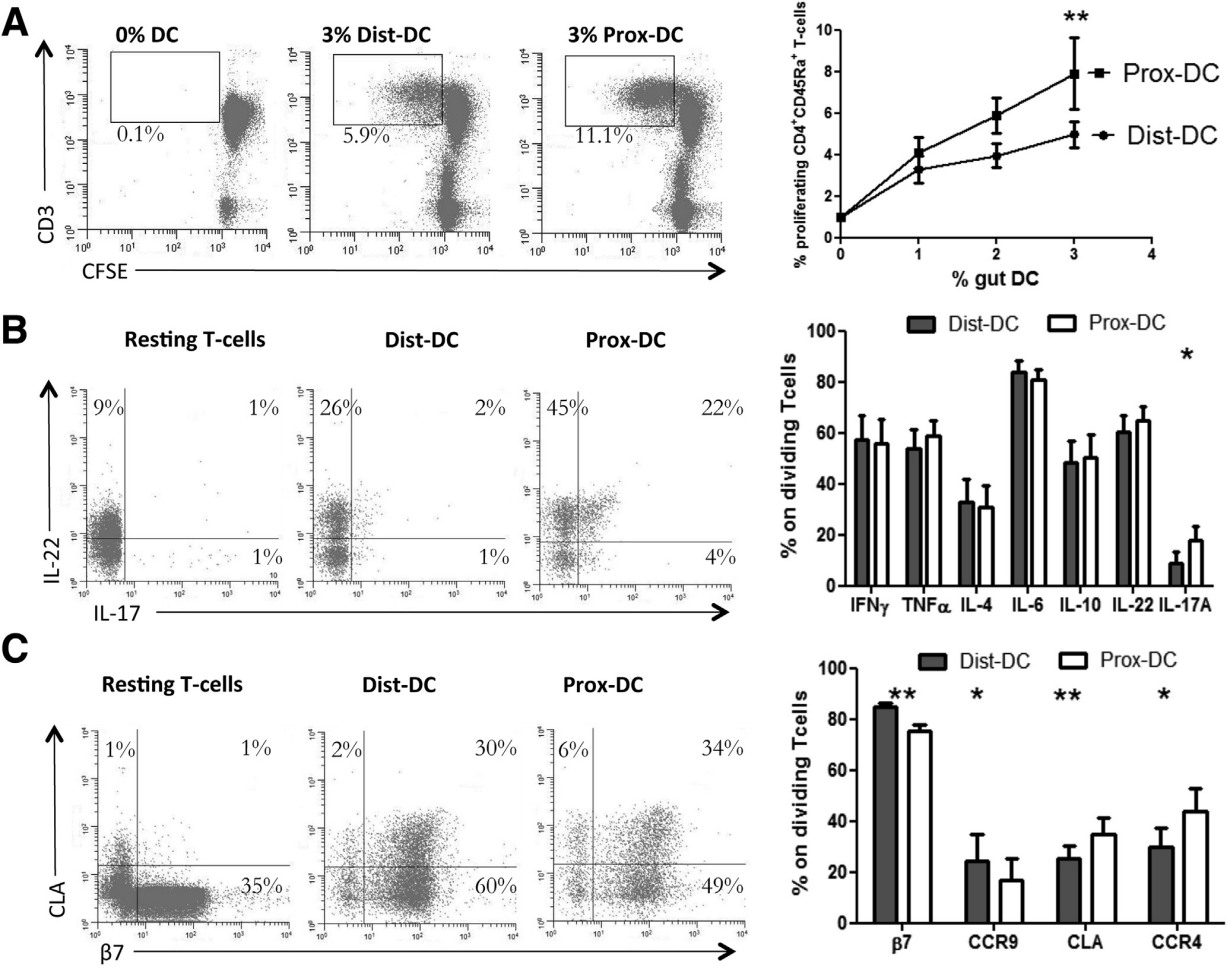
**Proximal colon dendritic cells (DC) are more stimulatory with lower gut-homing imprinting capacity than distal counterparts.** (*A*) After 400,000 CFSE-labeled allogeneic CD4^+^CD45RA^+^ T-cells were cultured with different numbers of paired proximal (Prox) and distal (Dist) human colonic DC, their stimulatory capacity was assessed as the percentage of proliferating T-cells (CD3^+^CFSE^low^) by flow cytometry. (*B*) Cytokine and (*C*) homing receptor profile of resting (nonstimulated) and dividing T cells (CD3^+^CFSE^low^) stimulated with 3% Prox/Dist colonic DC. Paired two-way analysis of variance (*A*) and paired *t* tests (*B*, *C*) were applied. *P* < .05 was considered statistically significant (*P < .05; **P < .01). Dot-plots are representative of eight independent experiments displayed in the graphics as the mean ± standard error.

Having described differences in the subsets and phenotype of proximal and distal colonic DC, we next studied whether these differences were reflected functionally. Colonic DC were enriched^21^, ^31^, ^33^ and cocultured with presorted CFSE-labeled allogeneic CD4^+^CD45RA^+^ T cells in a mixed leucocyte reaction (Figure 5*A*). Compared with blood DC, colonic DC display a low stimulatory capacity for naive CD4^+^ T cells,^31^ which was dose dependent. However, the distal colon DC stimulatory capacity was even lower than that of their proximal counterparts (see Figure 5*A*), correlating with their lower levels of maturation markers (see Figure 4*C*). Proximal DC induced a greater proportion of stimulated T cells to produce IL-17A, with no alterations in production of other measured cytokines (see Figure 5*B*). In agreement with lower homing marker/ligand expression in the proximal colon (see Figures 4*A* and *B*), proximal DC had lower GI-tract homing (β7 and CCR9) and higher non–GI-tract homing (cutaneous lymphocyte antigen [CLA] and CCR4) imprinting capacity for stimulated T cells (see Figure 5*C*). Thus, DC homing imprinting capacity may be not only tissue but also subtissue specific.

### Differences in the Proximal and Distal Colonic Microenvironments

**Figure 6:**
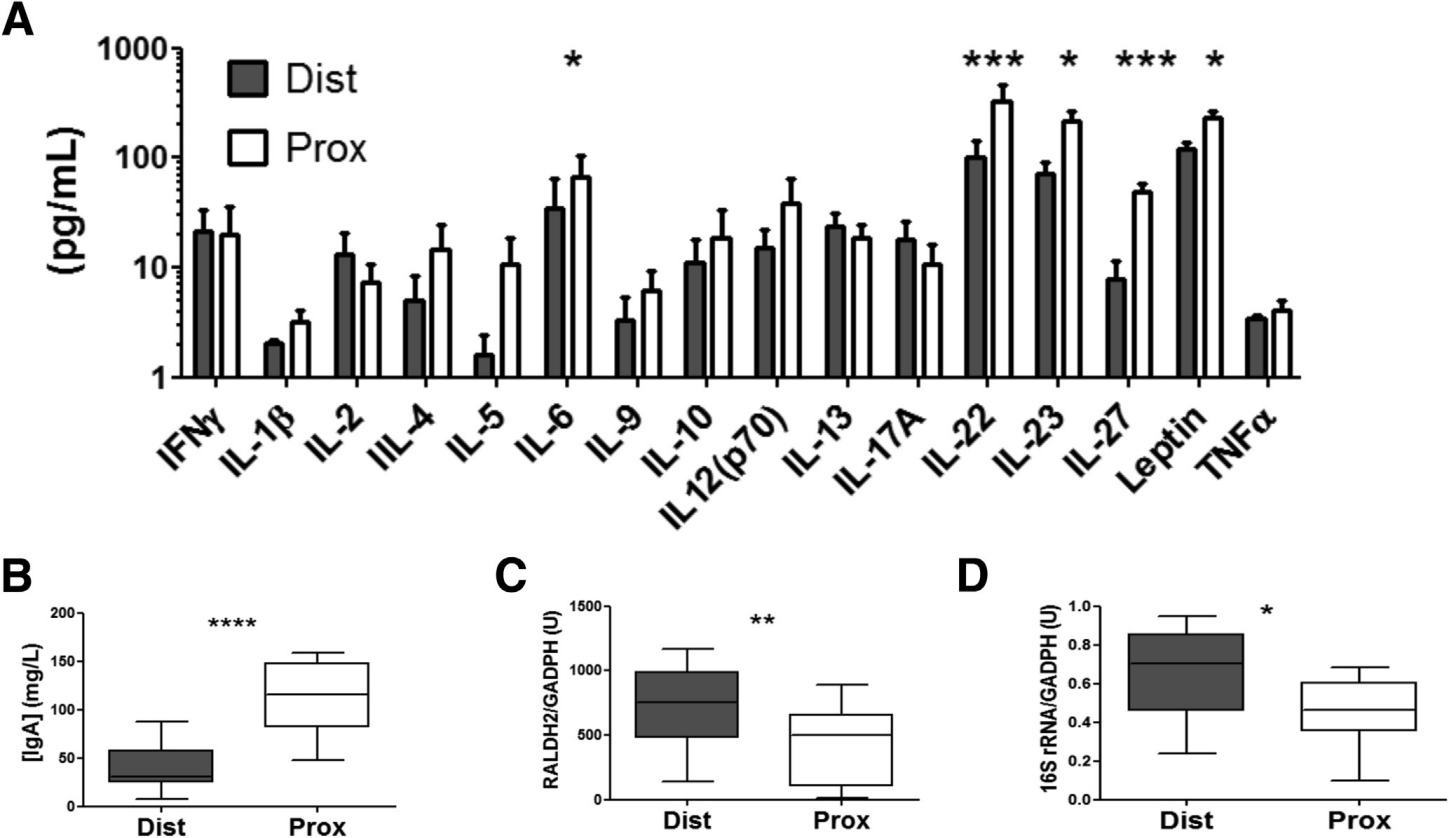

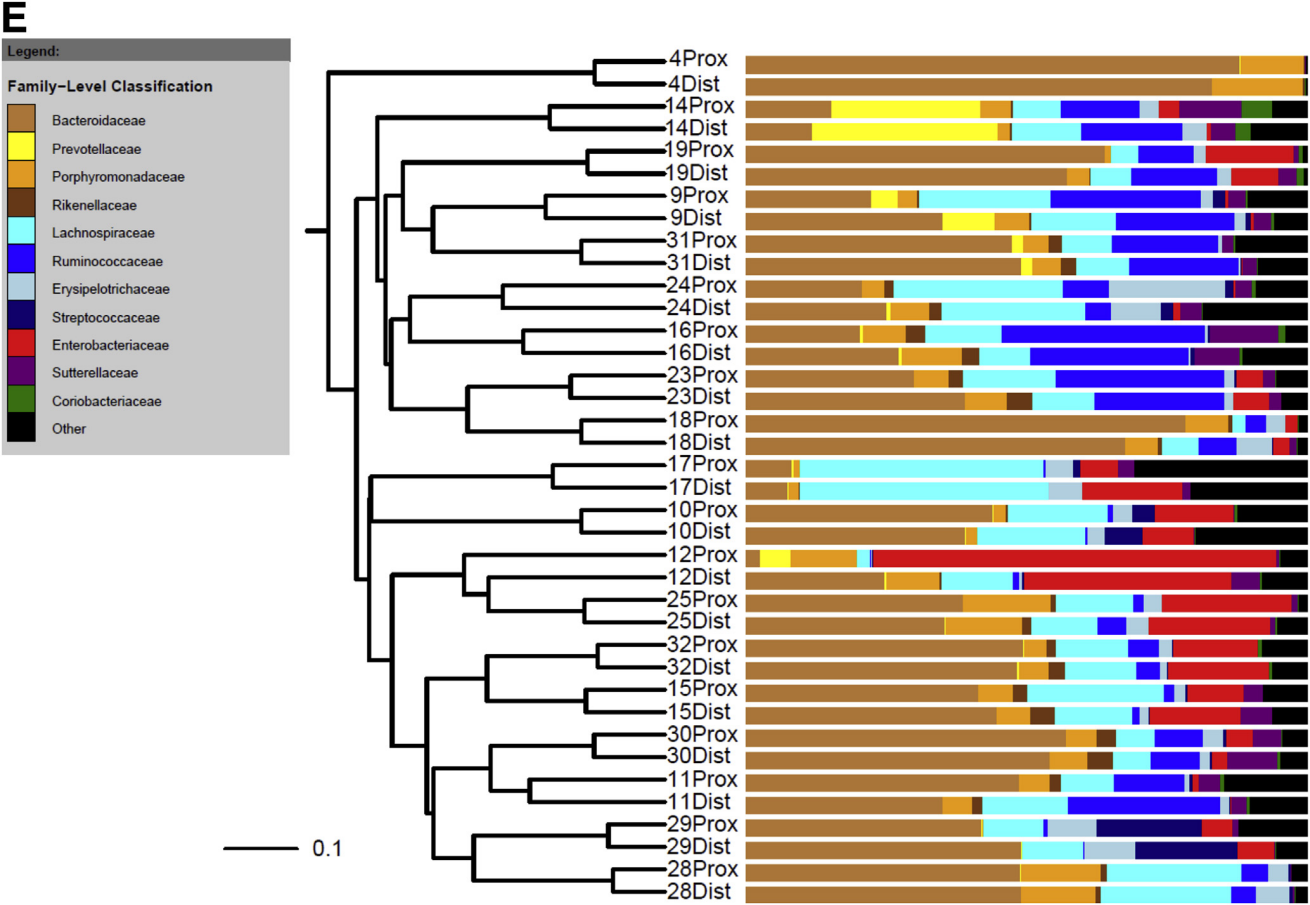
**Differences in the proximal and distal colonic microenvironments.** (*A*) Fresh paired biopsies samples from the distal (Dist) and proximal (Prox) human colon were cultured for 24 hours in complete medium and the cell-free culture supernatants assessed for soluble cytokines/adipokines by Multiplex and (*B*) IgA content by radial immunodiffusion. (*C*) RALDH2 mRNA expression was determined on noncultured biopsy tissues by quantitative reverse-transcription polymerase chain reaction (qRT-PCR), with the results displayed in arbitrary units (U). (*D*) Microbiota load, as measured by 16S rRNA quantity, was determined on noncultured biopsy tissues from the proximal (Prox) and distal (Dist) human colon by qRT-PCR, with the results displayed in arbitrary units (U). Paired *t* tests were applied, and *P* < .05 was considered statistically significant (*P < .05). (*E*) Cluster dendrogram, generated using the Bray Curtis calculator, displaying mucosal-associated microbiota compositional profiles in the human colonic biopsy tissues. Each pair of samples clusters together in the dendrogram, illustrating the large degree of interindividual variation rather than a signature proximal versus distal colonic bacterial profile. Bacterial families coloured in brown/yellow/orange represent the Bacteroidetes phylum, blue/grey the Firmicutes phylum, red/purple the Proteobacteria phylum, and green the Actinobacteria phylum. (*A*–*D*) Paired *t* tests, with *P* < .05 was considered statistically significant (*P < .05; **P < .01; ***P < .001; ****P < .0001). Results from *A*–*D* are displayed as mean ± standard error of 12 independent experiments.

Finally, we studied potential mechanisms that may account for differences between proximal and distal colon DC. Given that there was no differential capacity elicited by the proximal or distal human colon to recruit circulating DC (see Figure 3*F*), we next studied baseline levels of production of soluble immune mediators after in vitro culture of colonic biopsies. The proximal samples produced significantly larger amounts of IL-6, IL-22, IL-23, IL-27, leptin (Figure 6*A*), and IgA (see Figure 6*B*). The proximal colon also had lower levels of RALDH2 mRNA expression (see Figure 6*C*).

We also studied the colonic microbiota, as its activity is known to change through the length of the human GI tract.^6^, ^47^ We found that the overall mucosa-associated microbiota load was higher in the distal colon (see Figure 6*D*) although 16S rRNA gene sequencing analysis revealed no differences in mucosa-associated microbiota composition between the proximal and distal colonic mucosae (see Figure 6*E*). Mean Shannon Diversity Index scores, parsimony, and AMOVA tests comparing overall diversity and bacterial community structures between proximal/distal colon showed no significant differences, and there were no operational taxonomic units (OTUs) or taxonomic groups associated with particular colonic regions. Interindividual variation was the greatest driver of microbiota clustering patterns (see Figure 6*E*). These results are consistent with many previous studies describing the predominant mucosa-associated bacterial community as host specific and uniformly distributed along the colon while being significantly different from the fecal/luminal community.^48^, ^49^

## Discussion

Here, we report that human DC subsets, phenotype, and function differed between the proximal (right or ascending) and distal (left or descending) ends of the colon. Our results revealed that the proximal colon contained higher numbers of DC, which were immunologically more active. Thus, DC of the proximal colon showed a specific reduction of CD103^+^SIRPα^+^ DC and were typically more mature, with higher stimulatory capacity for T cells. However, balancing this increased stimulatory capacity, they had a lower potential to focus immune activity back to the colon, with lower expression of GI-tract homing markers β7 and CCR9, lower mRNA tissue expression of their ligands, and lower imprinting capacity of these markers on T cells they stimulate. Our results also revealed that intestinal CD103^+/−^SIRPα^+^ DC were similar to circulating CD1c^+^ mDC^9^ and may enter the colonic mucosa in a CCR2-dependent manner.

Human colon DC were divided into different subsets based on expression of CD103 and SIRPα, with the latter recently redefined as human analogs of murine CD11b.^9^, ^21^ Although murine CD103^−^CD11b^+^ DC can express intermediate levels of CX3CR1 (although less than in Mφ), CX3CR1 was absent in all human colon DC (Figure 2*E*).^17^ CD103 (αE) was coexpressed with β7 on DC (Figure 2*E*), with their abundance being reduced in the proximal colon, in agreement with the lower e-cadherin mRNA expression (ligand for CD103^+^β7^+^) (see Figure 4*A*) in that compartment. Differential CD103^+^ DC distribution may account for differences in colonic DC function as murine CD103^−^ DC have increased IL-17 priming capacity,^15^ as seen here among proximal human colonic DC (Figure 5*B*). SIRPα was equally expressed on proximal and distal colon DC, rendering the proximal colon with a specific reduction of CD103^+^SIRPα^+^. Further analysis confirmed that colonic CD103^+^SIRPα^+^ and CD103^-^SIRPα^+^ DC are CD1c^+^ whereas CD103^+^SIRPα^−^ are mainly CD141^+^, in agreement with recent observations relating intestinal SIRPα^+^ (both CD103^+^ and CD103^−^) to blood CD1c^+^ mDC and intestinal CD103^+^SIRPα^−^ to blood CD141^+^ mDC.^9^, ^10^ Indeed, both circulating CD1c^+^ mDC and intestinal SIRPα^+^ were CD1c^+^SIRPα^+^ILT3^+^CCR2^+^ whereas circulating CD141^+^ mDC and intestinal CD103^+^SIRPα^−^ were CD141c^+^SIRPα^-^ILT3^−^CCR2^−^ (see Figures 2 and 3).

Moreover, circulating CD1c^+^ mDC were recruited by human colonic culture supernatants in a CCR2-dependent manner (see Figure 3*F*). Given that DC are thought to acquire CD103 once they have entered the mucosa^10^ in a RALDH2-dependent manner,^50^ it may be possible that circulating CD1c^+^ mDC enter the mucosa as CD103^−^SIRPα^+^ DC via CCR2 and subsequently acquire CD103 at the time that they lose CCR2, thus explaining its variable expression on CD103^+^SIRPα^+^ DC (see Figure 3*A* and *B*). The lower expression of intestinal RALDH2 found in the proximal colon (see Figure 6*C*) may also explain the lower proportion of CD103^+^ DC in that compartment (see Figure 2*B* and *C*). The additional mechanisms responsible, however, for circulating CD141^+^ mDC recruitment by the human colon remain elusive, as they do not express CCR2; it is likely that other chemokine receptors may be involved in their recruitment.

**Supplementary Figure 1:**
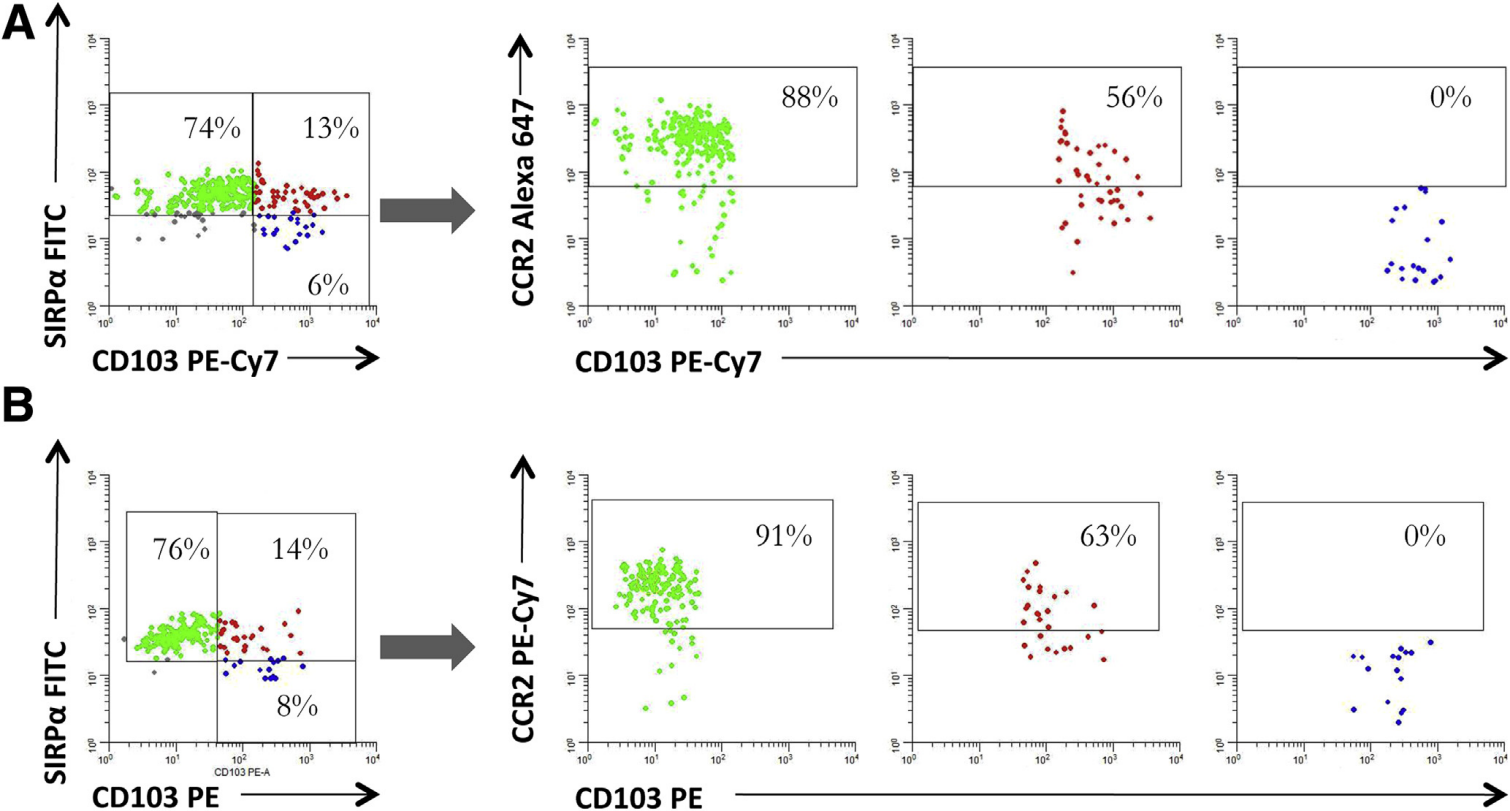
**Dendritic cells (DC) were identified as in**Figure 1***A* and divided into subsets based on expression of CD103 and SIRPα.** Chemokine (C-C motif) receptor 2 (CCR2) expression was determined in each DC subset using two different clones labeled with different fluorochromes: (*A*) clone 48607 labeled in Alexa 647 and (*B*) clone K036C2 labeled in PE-Cy7. The results are representative of two independent experiments performed with similar results.

Scott et al^20^ recently reported that intestinal CCR2^+^ DC were restricted to a fraction of CD103^−^SIRPα^+^ DC (both human and murine), with its expression being absent on the other intestinal subsets. However, we found that CCR2 was expressed by all CD103^-^SIRPα^+^, with expression being variable on CD103^+^SIRPα^+^ and absent on CD103^+^SIRPα^−^ DC. Further experiments confirmed these results using different CCR2 antibodies (Supplementary Figure 1), potentially eliminating a technical issue. One of the reasons for this discrepancy might be that Scott et al^20^ originally described CCR2^+^CD103^−^SIRPα^+^ DC in murine cells and confirmed their presence in human samples using tissue resections from patients with colorectal cancer. In contrast, in this study we used colonic biopsy samples from healthy intestines. Despite providing limited material, this use of normal tissue mitigates the abnormal effects of disease and treatment that are inherent when using resected tissue, typically derived from patients with acute GI cancer, inflammatory bowel diseases, and/or related diseases. All our samples were obtained from patients referred to clinics for changes in bowel transit, rectal bleeding, and/or screening for GI diseases, although in all cases they had normal tissue structure which was thus considered to provide normal, healthy colonic samples. Comparisons between healthy biopsy tissues and cancer resections would be required to test whether these differences in tissue source are the reason for different results.

DC acquire a “tolerogenic” profile after exposure to soluble factors derived from intestinal epithelial cells,^50^ and the proinflammatory milieu in inflammatory bowel disease may abrogate their function.^32^, ^51^ The proximal colon produced significantly larger amounts of soluble immune mediators (see Figures 6*A* and *B*), which may be responsible, at least to some extent, for the higher maturation status of proximal DC. RALDH2 promotes conversion of dietary vitamin A into retinoic acid, which mediates several of the tolerogenic properties of GI DC, including their capacity to generate Tregs and induce GI-tract homing markers on T cells they stimulate.^9^, ^10^, ^11^, ^12^, ^13^ Given that RALDH2 expression was lower in the proximal colon (see Figure 6*C*), and that retinoic acid mediates acquisition of GI-tract homing markers on immune cells, this may also account for the lower expression of GI-tract homing markers on DC and their lower imprinting capacity on T cells they stimulate. Lower GI-tract homing expression/imprinting capacity of proximal DC is also coupled with lower mRNA tissue expression of their ligands in the proximal colon (see Figure 4*A*). Therefore, it may be possible that the proximal and distal ends of the colon may use different levels of homing marker/ligand molecules to ensure compartmentalization of immune responses.

**Supplementary Figure 2:**
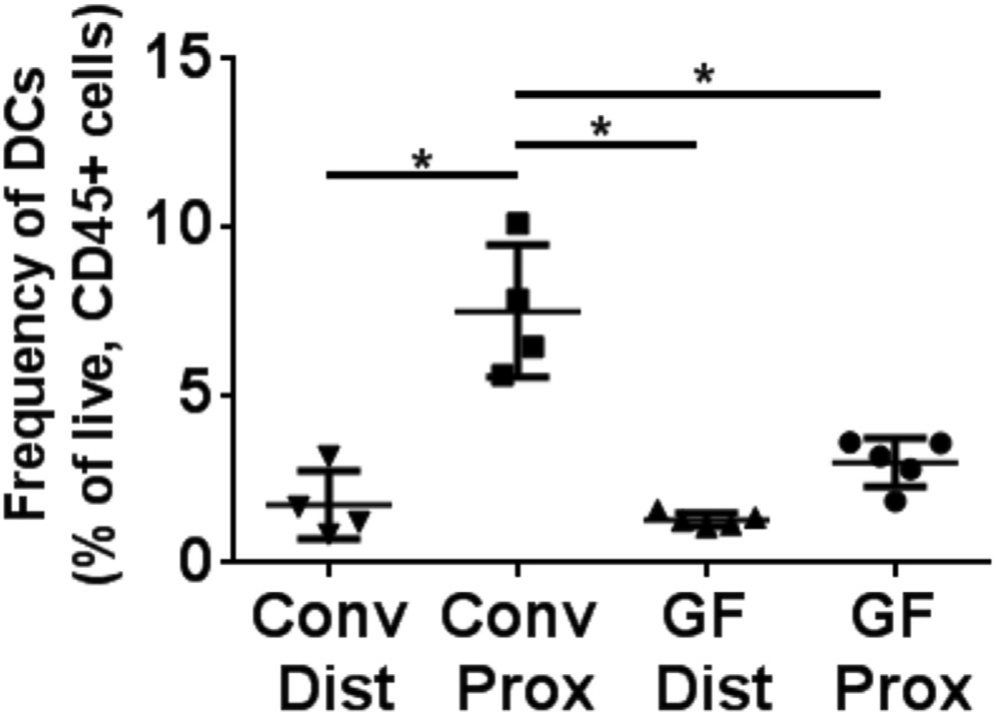
**Dendritic cell (DC) compartmentalization in the murine colon is modulated by the microbiota.** DC from proximal (Prox) and distal (Dist) regions of the colon in conventional (Conv) and germ-free (GF) mice were identified by flow cytometry within single colonic viable cells (ZombieAQUA^−^CD45^+^) and mean frequencies (with standard deviation) displayed. The results are representative of two independent experiments (n = 5 mice per group in each experiment). One-way analysis of variance with Tukey’s multiple comparisons was applied, and *P* < .05 was considered statistically significant (*P < .05, pair-wise comparisons without an asterisk were not statistically significant).

In addition to the differences in the cytokine milieu between the proximal and distal human colon, a differential modulatory effect of the colonic microbiota on intestinal DC may contribute. Indeed, the mucosa-associated microbiota load was lower in the proximal colon (see Figure 6*D*).^47^ Despite the similarity in mucosal microbiota composition between the proximal and distal colon (see Figure 6*E*), in agreement with other observations,^48^, ^49^ the metabolic output of the microbiota changes through the length of the GI tract as dietary compounds are gradually depleted by the microbiota as they pass through the colon.^26^ Therefore, the proximal and distal colonic microbiota produce different levels of short-chain fatty acids^52^, ^53^ and/or other extracellular molecular effectors,^54^ which may modulate DC properties and/or mucosal production of immune mediators (see Figures 6*A*–*C*). Indeed, we have shown that, similar to the human proximal/distal differences reported here, the frequency of DC was higher in the proximal colon of conventionally raised mice, with no differences between proximal and distal compartments in germ-free animals (Supplementary Figure 2; See Supplementary Materials and Methods for further details on the experiment). Therefore, regional DC differences throughout the colon might not only be influenced by the cytokine milieu itself but also directly or indirectly by members of the microbiota.

In summary, our data suggest that human blood CD1c^+^ mDC are recruited by the colonic mucosa in a CCR2-dependent manner and confirm that DC subsets, phenotype, and function are different between the proximal and distal human colon, and reveal the proximal colon DC as potentially more immunologically active than their distal counterparts. Studies of the immune system of the GI tract need therefore to take into account the immune compartmentalization throughout its length. Future studies will address whether GI-tract DC compartmentalization contributes to diseases such as subtypes of inflammatory bowel diseases and/or colorectal cancer where disease is anatomically restricted to different GI locations. Differences in disease incidence and severity could therefore be related to the varying immune activity of different GI regions.

## Figures and Tables

**Supplementary Table 1:** Antibodies and Flow Cytometry

Antibody Specificity	Clone	Conjugate	Manufacturer
β7	FIB504	PE	BD Biosciences
CCR2	K036C2	PE-Cy7	BioLegend
	K036C2	APC	BioLegend
	48607	Alexa 647	BD Biosciences
CCR4	205410	APC	R&D Systems
CCR7	150503	PE	R&D Systems
CCR8	191704	APC	R&D Systems
CCR9	248621	PE	R&D Systems
CCR10	314305	APC	R&D Systems
CD1c (BDCA1)	AD5–8E7	FITC	Miltenyi Biotech
	AD5–8E7	PE	Miltenyi Biotech
	L161	Alexa700	BioLegend
CD3	UCHT1	PE-Cy5	BD Biosciences
CD11c	KB90	FITC	Dako
	B-Ly6	PE	BD Biosciences
	3.9	PE-Cy7	eBioscience
	B-Ly6	BV605	BD Biosciences
	Bu15	APC-Cy7	BioLegend
CD14	M5E2	PE	BD Biosciences
	TÜK4	PE-Cy5	AbD Serotec
	MΦP9	PE-CF594	BD Biosciences
CD16	3G8	PE-Cy5	BD Biosciences
CD19	HIB19	PE-Cy5	BD Biosciences
CD34	581	PE-Cy5	BD Biosciences
CD40	LOB7/6	FITC	BD Biosciences
	LOB7/6	PE	BD Biosciences
CD45	HI30	PE-Cy7	BD Biosciences
	VS143	APC-Cy7	BioLegend
	HI30	PE-Cy7	BioLegend
	HI30	BUV395	BD Biosciences
CD64	10.1	PE-Cy5	Abcam
	10.1	PE-Cy7	BioLegend
CD80	L307.4	FITC	BD Biosciences
	L307.4	PE	BD Biosciences
CD86	BU63	FITC	AbD Serotec
CD103	Ber-ACT8	FITC	BD Biosciences
	Ber-ACT8	PE	BioLegend
	Ber-ACT8	BV421	BD Biosciences
	Ber-ACT8	PE-Cy7	BioLegend
CD123	6H6	PE-Cy7	eBioscience
	7G3	BV786	BD Biosciences
CD141 (BDCA3)	501733	FITC	R&D Systems
	1A4	BV711	BD Biosciences
	AD5–14H12	PE	Miltenyi Biotech
CD172a (SIRPα)	602411	FITC	R&D Systems
	602411	PE	R&D Systems
	SE5A5	PE-Cy7	BioLegend
CD209 (DC-SIGN)	120507	FITC	R&D Systems
	120507	PE	R&D Systems
CD303 (BDCA2)	AC144	FITC	Miltenyi Biotech
CLA	HECA-452	APC	BD Biosciences
	HECA-452	Biotin	BD Biosciences
CX3CR1	528728	PE	R&D Systems
HLA-DR	G46–6	APC	R&D Systems
	L243	BV570	BioLegend
IFNγ	25723.11	APC	BD Biosciences
IL-4	8DA-8	PE-Cy7	eBioscience
IL-6	AS12	FITC	eBioscience
	1936	PE	R&D Systems
IL-10	BT-10	FITC	eBioscience
	JES3–9D7	PE-Cy7	eBioscience
	JES3–19F1	APC	BD Biosciences
IL-17A	eBIO64DEC17	APC	eBioscience
IL-22	22URTI	APC	eBioscience
	IL22JOP	PE-Cy7	eBioscience
ILT3	293623	PE	R&D Systems
Streptavidin	not applied	APC	BD Biosciences
TGFβ	35409	PE	R&D Systems
TLR2	TLR2.3	FITC	AbD Serotec
TLR4	HTA125	FITC	AbD Serotec
TNFα	B-D9	FITC	AbD Serotec
	Mab11	PE-Cy7	eBioscience

Note: AbD Serotec, Kidlington, United Kingdom; BD Biosciences, Oxford, United Kingdom; BioLegend, San Diego, CA; Dako, Glostrup, Denmark; eBioscience, Wien, Austria; Miltenyi Biotech, Bisley, United Kingdom; R&D Systems, Minneapolis, MN.

**Supplementary Table 2:** Quantitative Polymerase Chain Reaction Primers

Molecule	Primers Sequence	Ta	Primer Source
GADPH			
Forward	5′-GAAGGTGAAGGTCGGAGTC-3′	60	Ren et al, 2005
Reverse	5′-GAAGATGGTGATGGGATTTC-3′		
16S rRNA			
Forward	5′-TTAAACTCAAAGGAATTGACGG-3′	68	Ozbek et al, 2009
Reverse	5′-CTCACGRCACGAGCTGACGAC-3′		
RALDH2	5532957001_Roche	60	Roche Diagnostics
CCL25			
Forward	5′-GATAAAACCGTCGCCCTACA-3′	58	NCBI Reference Sequence: NM_005624.3
Reverse	5′-TCCTTTGGGTCTGCACATAGC-3′		
MADCAM1			
Forward	5′-CTGTACGGCCCACAAAGTCA-3′	60	NCBI Reference Sequence: NM_130760.2
Reverse	5′-TCTGTCACCCTGAACAGCAC-3′		
e-cadherin			
Forward	5′-CAGTCTCTTCTCTCACGCGT-3′	60	NCBI Reference Sequence: NM_004360.3
Reverse	5′-TGAGGATGGTGTAAGCGATGG-3′		

Note: Primers used for quantitative polymerase chain reaction, including primer sequence or source and annealing temperature (Ta). Primers for CCL25, MADCAM1, and e-cadherin were designed with Primer-BLAST (http://www.ncbi.nlm.nih.gov/tools/primer-blast/) using the reference sequences provided in the table.

*Sources:* Ren M, Pozzi S, Bistulfi G, et al. Impaired retinoic acid (RA) signal leads to RARβ2 epigenetic silencing and RA resistance. Mol Cell Biol 2005;25:10591–10603; Ozbek SM, Ozbek A, Erdorgan AS. Analysis of *Enterococcus faecalis* in samples from Turkish patients with primary endodontic infections and failed endodontic treatment by real-time PCR SYBR green method. J Appl Oral Sci 2009;17:370–374; Ye J, Coulouris G, Zaretskaya I, et al. Primer-BLAST: a tool to design target-specific primers for polymerase chain reaction. BMC Bioinformatics 2012;13:134.

**Supplementary Table 3:** European Nucleotide Archive (ENA) Accession Number Information for Each of the Biopsy Tissue Sample Sequencing Results From the Microbiota Analyses

Sample Name	Biopsy Sample Info	ENA Sample Accession No.
4L	Patient 4, distal colon	ERS557964
4R	Patient 4, proximal colon	ERS557965
9L	Patient 9, distal colon	ERS557966
9R	Patient 9, proximal colon	ERS557967
10L	Patient 10, distal colon	ERS557968
10R	Patient 10, proximal colon	ERS557969
11L	Patient 11, distal colon	ERS557970
11R	Patient 11, proximal colon	ERS557971
12L	Patient 12, distal colon	ERS557972
12R	Patient 12, proximal colon	ERS557973
14L	Patient 14, distal colon	ERS557974
14R	Patient 14, proximal colon	ERS557975
15L	Patient 15, distal colon	ERS557976
15R	Patient 15, proximal colon	ERS557977
16L	Patient 16, distal colon	ERS557978
16R	Patient 16, proximal colon	ERS557979
17L	Patient 17, distal colon	ERS557980
17R	Patient 17, proximal colon	ERS557981
18L	Patient 18, distal colon	ERS557982
18R	Patient 18, proximal colon	ERS557983
19L	Patient 19, distal colon	ERS557984
19R	Patient 19, proximal colon	ERS557985
23L	Patient 23, distal colon	ERS557986
23R	Patient 23, proximal colon	ERS557987
24L	Patient 24, distal colon	ERS557988
24R	Patient 24, proximal colon	ERS557989
25L	Patient 25, distal colon	ERS557990
25R	Patient 25, proximal colon	ERS557991
28L	Patient 28, distal colon	ERS557992
28R	Patient 28, proximal colon	ERS557993
29L	Patient 29, distal colon	ERS557994
29R	Patient 29, proximal colon	ERS557995
30L	Patient 30, distal colon	ERS557996
30R	Patient 30, proximal colon	ERS557997
31L	Patient 31, distal colon	ERS557998
31R	Patient 31, proximal colon	ERS557999
32L	Patient 32, distal colon	ERS558000
32R	Patient 32, proximal colon	ERS558001

Note: Samples marked L are from the left or distal colon; those marked R are from the right or proximal colon. European Nucleotide Archive (http://www.ebi.ac.uk/ena).

## Supplementary Materials and Methods

### Murine Experiments

Conventionally raised and germ-free (reared from germ-free breeding pairs) male C57BL/6 mice were sacrificed at 8 weeks of age, and lamina propria cells were isolated for flow cytometry analysis. Briefly; proximal and distal large intestine was chopped into small pieces and incubated with vigorous stirring (200 rpm) in complete media with 2 mM EDTA for 15 minutes at 37°C. This was repeated, then colon pieces were recovered and incubated for 40 minutes at 37°C with collagenase (type VIII, 100 U/mL; Sigma-Aldrich). The lamina propria cells were then filtered, washed, and stained for flow cytometry analysis on a BD Fortessa instrument (BD Biosciences, Oxford, United Kingdom) and FlowJo software (Treestar, Ashland, OR). Antibodies used were CD45 (clone 20-F11; BioLegend, San Diego, CA), MHCII (clone 2G9; BD Biosciences), and CD11c (clone N418; eBioscience, Wien, Austria). Staining was performed at 4°C in the presence of Fc receptor block (anti-CD16/32, clone 93; BioLegend). Zombie AQUA (BioLegend) was used for live-dead discrimination according to manufacturer’s directions. All experiments were performed with United Kingdom Home Office approval.

## References

[1] Stagg A.J., Kamm M.A., Knight S.C. (2002). Intestinal dendritic cells increase T cell expression of α4β7 integrin. Eur J Immunol.

[2] Stagg A.J., Hart A.L., Knight S.C. (2003). The dendritic cell: its role in intestinal inflammation and relationship with gut bacteria. Gut.

[3] Mowat A.M. (2003). Anatomical basis of tolerance and immunity to intestinal antigens. Nat Rev Immunol.

[4] Chirdo F.G., Millington O.R., Beacock-Sharp H. (2005). Immunomodulatory dendritic cells in intestinal lamina propria. Eur J Immunol.

[5] Collin M., Bigley V., Haniffa M. (2011). Human dendritic cell deficiency: the missing ID?. Nat Rev Immunol.

[6] Bogunovic M., Ginhoux F., Helft J. (2009). Origin of the lamina propria dendritic cell network. Immunity.

[7] Schulz O., Jaensson E., Persson E.K. (2009). Intestinal CD103+, but not CX3CR1^+^, antigen sampling cells migrate in lymph and serve classical dendritic cell functions. J Exp Med.

[8] Varol C., Vallon-Eberhard A., Elinav E. (2009). Intestinal lamina propria dendritic cell subsets have different origin and functions. Immunity.

[9] Watchmaker P.B., Lahl K., Lee M. (2014). Comparative transcriptional and functional profiling defines conserved programs of intestinal DC differentiation in humans and mice. Nat Immunol.

[10] Bekiaris V., Persson E.K., Agace W.W. (2014). Intestinal dendritic cells in the regulation of mucosal immunity. Immunol Rev.

[11] Scott C.L., Aumeunier A.M., Mowat A.M. (2011). Intestinal CD103^+^ dendritic cells: master regulators of tolerance?. Trends Immunol.

[12] Kinnebrew M.A., Buffie C.G., Diehl G.E. (2012). Interleukin 23 production by intestinal CD103^+^CD11b^+^ dendritic cells in response to bacterial flagellin enhances mucosal innate immune defense. Immunity.

[13] Persson E.K., Scott C.L., Mowat A.M. (2013). Dendritic cell subsets in the intestinal lamina propria: ontogeny and function. Eur J Immunol.

[14] Persson E.K., Uronen-Hansson H., Semmrich M. (2013). IRF4 transcription-factor-dependent CD103^+^CD11b^+^ dendritic cells drive mucosal T helper 17 cell differentiation. Immunity.

[15] Cerovic V., Houston S.A., Scott C.L. (2013). Intestinal CD103^−^ dendritic cells migrate in lymph and prime effector T cells. Mucosal Immunol.

[16] Gibbons D.L., Spencer J. (2011). Mouse and human intestinal immunity: same ballpark, different players; different rules, same score. Mucosal Immunol.

[17] Mann E.R., Landy J.D., Bernardo D. (2013). Intestinal dendritic cells: their role in intestinal inflammation, manipulation by the gut microbiota and differences between mice and men. Immunol Lett.

[18] Sanders T.J., McCarthy N.E., Giles E.M. (2014). Increased production of retinoic acid by intestinal macrophages contributes to their inflammatory phenotype in patients with Crohn’s disease. Gastroenterology.

[19] Denning T.L., Norris B.A., Medina-Contreras O. (2011). Functional specializations of intestinal dendritic cell and macrophage subsets that control Th17 and regulatory T cell responses are dependent on the T cell/APC ratio, source of mouse strain, and regional localization. J Immunol.

[20] Scott C.L., Bain C.C., Wright P.B. (2015). CCR2^+^CD103^−^ intestinal dendritic cells develop from DC-committed precursors and induce interleukin-17 production by T cells. Mucosal Immunol.

[21] Mann E.R., Bernardo D., English N.R. (2015). Compartment-specific immunity in the human gut: properties and functions of dendritic cells in the colon versus the ileum. Gut.

[22] Mowat A.M., Agace W.W. (2014). Regional specialization within the intestinal immune system. Nat Rev Immunol.

[23] Kaz A.M., Wong C.J., Dzieciatkowski S. (2014). Patterns of DNA methylation in the normal colon vary by anatomical location, gender, and age. Epigenetics.

[24] Eriksson K., Quiding-Jarbrink M., Osek J. (1999). Anatomic segmentation of the intestinal immune response in nonhuman primates: differential distribution of B cells after oral and rectal immunizations to sites defined by their source of vascularization. Infect Immun.

[25] Glebov O.K., Rodriguez L.M., Nakahara K. (2003). Distinguishing right from left colon by the pattern of gene expression. Cancer Epidemiol Biomarkers Prev.

[26] Cummings J.H., Macfarlane G.T. (1991). The control and consequences of bacterial fermentation in the human colon. J Appl Bacteriol.

[27] Bauer K.M., Hummon A.B., Buechler S. (2012). Right-side and left-side colon cancer follow different pathways to relapse. Mol Carcinog.

[28] Harrell L., Wang Y., Antonopoulos D. (2012). Standard colonic lavage alters the natural state of mucosal-associated microbiota in the human colon. PLoS One.

[29] Mai V., Greenwald B., Morris J.G. (2006). Effect of bowel preparation and colonoscopy on post-procedure intestinal microbiota composition. Gut.

[30] Hart A.L., Al-Hassi H.O., Rigby R.J. (2005). Characteristics of intestinal dendritic cells in inflammatory bowel diseases. Gastroenterology.

[31] Mann E.R., Bernardo D., Al-Hassi H.O. (2012). Human gut-specific homeostatic dendritic cells are generated from blood precursors by the gut microenvironment. Inflamm Bowel Dis.

[32] Al-Hassi H.O., Bernardo D., Murugananthan A.U. (2013). A mechanistic role for leptin in human dendritic cell migration: differences between ileum and colon in health and Crohn’s disease. Mucosal Immunol.

[33] Bell S.J., Rigby R., English N. (2001). Migration and maturation of human colonic dendritic cells. J Immunol.

[34] Al-Hassi H.O., Mann E.R., Sanchez B. (2014). Altered human gut dendritic cell properties in ulcerative colitis are reversed by *Lactobacillus plantarum* extracellular encrypted peptide STp. Mol Nutr Food Res.

[35] Maroof A., English N.R., Bedford P.A. (2005). Developing dendritic cells become ‘lacy’ cells packed with fat and glycogen. Immunology.

[36] Patterson S., Gross J., Bedford P. (1991). Morphology and phenotype of dendritic cells from peripheral blood and their productive and non-productive infection with human immunodeficiency virus type 1. Immunology.

[37] Kozich J.J., Westcott S.L., Baxter N.T. (2013). Development of a dual-index sequencing strategy and curation pipeline for analyzing amplicon sequence data on the MiSeq Illumina sequencing platform. Appl Environ Microbiol.

[38] Quince C., Lanzen A., Davenport R.J. (2011). Removing noise from pyrosequenced amplicons. BMC Bioinformatics.

[39] Letunic I., Bork P. (2011). Interactive Tree of Life v2: online annotation and display of phylogenetic trees made easy. Nucleic Acids Res.

[40] White J.R., Nagarajan N., Pop M. (2009). Statistical methods for detecting differentially abundant features in clinical metagenomic samples. PLoS Comput Biol.

[41] Segata N., Izard J., Waldron L. (2011). Metagenomic biomarker discovery and explanation. Genome Biol.

[42] Schloss P.D., Westcott S.L., Ryabin T. (2009). Introducing mothur: open-source, platform-independent, community-supported software for describing and comparing microbial communities. Appl Environ Microbiol.

[43] Raki M., Beitnes A.C., Lundin K.E. (2013). Plasmacytoid dendritic cells are scarcely represented in the human gut mucosa and are not recruited to the celiac lesion. Mucosal Immunol.

[44] Scott C.L., Murray T.F., Beckham K.S. (2014). Signal regulatory protein alpha (SIRPα) regulates the homeostasis of CD103 CD11b DCs in the intestinal lamina propria. Eur J Immunol.

[45] Chang C.C., Ciubotariu R., Manavalan J.S. (2002). Tolerization of dendritic cells by T(S) cells: the crucial role of inhibitory receptors ILT3 and ILT4. Nat Immunol.

[46] Beitnes A.C., Raki M., Brottveit M. (2012). Rapid accumulation of CD14^+^CD11c^+^ dendritic cells in gut mucosa of celiac disease after in vivo gluten challenge. PLoS One.

[47] Hayashi H., Takahashi R., Nishi T. (2005). Molecular analysis of jejunal, ileal, caecal and recto-sigmoidal human colonic microbiota using 16S rRNA gene libraries and terminal restriction fragment length polymorphism. J Med Microbiol.

[48] Lepage P., Seksik P., Sutren M. (2005). Biodiversity of the mucosa-associated microbiota is stable along the distal digestive tract in healthy individuals and patients with IBD. Inflamm Bowel Dis.

[49] Eckburg P.B., Bik E.M., Bernstein C.N. (2005). Diversity of the human intestinal microbial flora. Science.

[50] Iliev I.D., Spadoni I., Mileti E. (2009). Human intestinal epithelial cells promote the differentiation of tolerogenic dendritic cells. Gut.

[51] Bernardo D., Vallejo-Diez S., Mann E.R. (2012). IL-6 promotes immune responses in human ulcerative colitis and induces a skin-homing phenotype in the dendritic cells and T cells they stimulate. Eur J Immunol.

[52] Arpaia N., Campbell C., Fan X. (2013). Metabolites produced by commensal bacteria promote peripheral regulatory T-cell generation. Nature.

[53] Smith P.M., Howitt M.R., Panikov N. (2013). The microbial metabolites, short-chain fatty acids, regulate colonic Treg cell homeostasis. Science.

[54] Ruiz L., Hevia A., Bernardo D. (2014). Extracellular molecular effectors mediating probiotic attributes. FEMS Microbiol Lett.

